# Sustainable Solutions for Producing Advanced Biopolymer Membranes—From Net-Zero Technology to Zero Waste

**DOI:** 10.3390/polym17111432

**Published:** 2025-05-22

**Authors:** Iva Rezić Meštrović, Maja Somogyi Škoc, Donna Danijela Dragun, Petra Glagolić, Ernest Meštrović

**Affiliations:** 1 Faculty of Textile Technology, University of Zagreb, 10000 Zagreb, Croatia; maja.somogyi@ttf.unizg.hr; 2 Faculty of Chemical Engineering and Technology, University of Zagreb, 10000 Zagreb, Croatia; ddragun@fkit.hr (D.D.D.); pglagolic@fkit.hr (P.G.); emestrov@fkit.unizg.hr (E.M.)

**Keywords:** zero waste, zero-net technology, sustainable biopolymers, membranes, coffee-ring effect, life cycle assessment, electrospinning, optimization, design of experiment, AI tools

## Abstract

The increasing accumulation of polymer waste presents a significant environmental challenge and a critical opportunity for the development of circular and sustainable membranes. The answer to this complex topic requires an integral approach covering different aspects of the problem. This paper, therefore, explores innovative approaches for the chemical recycling of polymer waste into value-added products, with a specific emphasis on the production of advanced biopolymer membranes. By converting discarded materials into functional polymers through depolymerization and chemical modification processes, new pathways are emerging for the fabrication of high-performance membranes used in filtration, biomedical applications, and energy systems. Among these, electrospinning has gained prominence as a versatile and scalable technique for producing nanostructured membranes with tailored properties. As a key case study presented, the focus was on the optimization of electrospinning parameters, including solvents, polymer concentration, voltage, and flow rate, for the investigation of membranes derived from recycled materials to achieve net-zero technology. Moreover, the environmental benefits of this approach are discussed within a zero-waste and net-zero carbon framework, emphasizing the integration of life cycle assessment to evaluate sustainability metrics. This paper underscores the potential of polymer waste as a feedstock for circular membrane technologies and provides a roadmap for future innovations in waste-to-resource strategies. The results of the demonstrated case example clearly demonstrate how the effects of processing conditions on the production of fine-tuned biodegradable membranes with controlled porosity influenced membrane properties, including mechanical strength and surface functionality, for the desired suppression of the coffee-ring effect.

## 1. Introduction

The polymer and textile industries are two of the largest contributors to global waste and environmental degradation. Traditional disposal methods, such as landfilling and incineration, are no longer viable due to their ecological impact. As such, sustainable approaches to valorizing textile waste have become crucial. Among these, the chemical recycling of waste textiles into biopolymer membranes offers a dual advantage, reducing environmental burden and producing high-value functional materials.

Polymer and textile waste is an increasingly urgent environmental issue, with millions of tons discarded annually worldwide. There is a critical need for beyond State-of-the-Art technologies and strategies for transforming polymer waste into high-performance biopolymer membranes through chemical recycling, with a particular focus on State-of-the-Art techniques such as recycling and electrospinning for making advanced materials, taking into account all relevant environmental implications of textile or polymer waste, the chemistry of polymer recovery, and innovations in membrane fabrication.

The term *net-zero technology* refers to innovations that reduce or eliminate greenhouse gas emissions, ensuring that the amount of emissions released is balanced by the amount removed or offset. These technologies play a crucial role in achieving climate goals by enabling sustainable production and circular resource use. As an example, converting textile waste into high-performance membranes for water purification or energy applications for turning discarded materials into valuable, low-carbon products will be presented in this work. Moreover, “*zero-waste*” technology focuses on designing processes and products that eliminate waste by reusing, recycling, or repurposing materials in a closed-loop system, aiming at minimizing environmental impact by ensuring that all resources are fully utilized and no waste is discarded. In the example of transforming textile waste into advanced membranes for filtration or energy storage, giving new life to discarded fabrics while preventing landfill accumulation, zero-waste technology is explored.

A detailed case study on the optimization of electrospinning parameters is discussed to illustrate the potential for developing nanostructured membranes with controlled functionalities. This study integrates concepts of zero-waste manufacturing and net-zero carbon technologies, positioning polymer waste recycling as a cornerstone in sustainable materials science.

Zero-waste technology refers to a set of principles, strategies, and innovations aimed at eliminating waste generation across industrial and societal systems. It embodies the concept of designing products, processes, and systems in ways that ensure all resources are reused, recycled, or composted, with the ultimate goal of sending nothing to landfills, incinerators, or the environment.

At the heart of zero waste lies the circular economy model, which contrasts with the traditional linear approach of “take-make-dispose”. In a circular system, materials are kept in use for as long as possible through continuous recovery, reintegration, and regeneration. Zero-waste technologies facilitate this model by optimizing resource efficiency, minimizing pollution, and promoting sustainable product life cycles.

Key components of zero-waste technology include material recovery and upcycling, green design and eco-innovation, biotechnological and chemical advancements, and waste-to-resource systems. Such processes are needed in many industries, such as the polymer and textile industries. For example, only 1% of textile materials are effectively recycled into new clothing, with 12% recycled overall, leaving 88% of the estimated 92 million tonnes of textile waste generated globally each year unused [1]. This presents a major environmental challenge and leads to significant financial burdens for the textile industry. In the EU, the consumption of textiles, most of which are imported, now accounts, on average, for the fourth highest negative impact on the environment and climate change and the third highest for water and land use from a global life cycle perspective [2,3,4].

**Figure 1:**
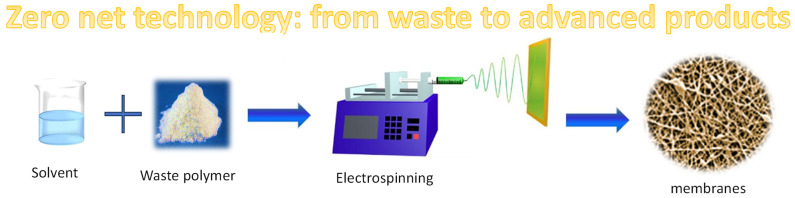
Concept of net-zero technology applied to recycled biopolymers to create advanced membrane systems.

Integrating the principles of green chemistry and circular economy to minimize waste, repurpose cellulose materials, and enhance the performance of filtration membranes by advancing them using a State-of-the-Art modeling approach is very promising (Figure 1).

Polymer waste comprises natural fibers (cotton and wool), synthetic polymers (polyester and nylon), and blended materials. Polyester, in particular, dominates global fiber production and contributes significantly to microplastic pollution. The unsustainable growth in polymer consumption, combined with ineffective waste management infrastructure, poses significant threats to ecosystems, human health, and the global economy. Despite current actions taken by the European Union, which range from improved recycling technologies to international policies on waste reduction and a strong focus on technological developments in the search for integral sustainable solutions, such methods need several years to obtain fruitful results, which are crucial to mitigate these escalating environmental impacts and transition toward a circular, zero-waste economy. A promising approach is using polymer waste to produce advanced materials. One of the most sustainable and scalable solutions is fiber-to-fiber recycling, transforming textile waste into new materials of added value. This field is evolving rapidly, driven by innovation and the push for scalability. While the mechanical recycling of pure cotton is already well-established, technologies such as chemical recycling with green solvents are still in the development stage. Once these technologies reach full maturity, it is estimated that up to 70% of textile waste could be fiber-to-fiber recycled, while the remaining 30% would require open-loop recycling or alternative processes like thermo-chemical conversion into syngas.

We will fill the gap in the existing technology by recycling regenerated waste cellulose polymers into biodegradable (nano)composite membranes.

Membranes are thin layers of material that act as barriers between two spaces, allowing the selective transfer of substances. In both nature and technology, membranes play a crucial role in various processes, from biological functions to industrial applications.

In biology, membranes are fundamental components of all living cells. The cell membrane (plasma membrane) surrounds each cell, protecting it and enabling controlled entry and exit of molecules such as water, ions, nutrients, and waste products. It is primarily composed of a lipid bilayer and embedded proteins that function as channels, receptors, or enzymes. In addition to the cell membrane, there are internal membranes, such as those of the mitochondria or endoplasmic reticulum, which organize and facilitate specialized processes within the cell. In the biological context, membranes can also be semipermeable, meaning they allow only specific molecules to pass through. This property is the basis for osmosis and diffusion, which are key to maintaining homeostasis in the body. In industry, membranes are used in numerous technologies, primarily for separation processes. Reverse osmosis and nanofiltration use membranes to purify water, desalinate seawater, or remove harmful substances from wastewater. Membrane filtration is also employed in the food industry, pharmaceuticals, and chemical production.

Technological membranes can be natural (e.g., cellulose-based) or synthetic, made from various polymers chosen according to their application. Key properties include permeability, selectivity, mechanical stability, and chemical resistance. There are also membranes for fuel cells that allow selective ion transfer, enabling the conversion of chemical energy into electrical energy. In medicine, artificial membranes are used for dialysis, acting as substitutes for kidney function by purifying a patient’s blood. Membranes are also vital research tools, especially in the fields of nanotechnology, biotechnology, and sensor development. Advances in membrane science are enabling the development of increasingly precise, efficient, and energy-saving separation and detection systems. Due to their versatility and significance across many scientific and engineering disciplines, membranes remain one of the most active areas of research and application in modern science.

Nanocomposite technology offers a promising solution to this challenge, as the integration of antimicrobial nanoparticles can enhance membrane performance by preventing microbial growth, thereby extending membrane lifespan and improving filtration efficiency. In order to be sustainable, membranes should be biodegradable.

Biodegradation is the breakdown of matter into smaller parts and eventual conversion into substances that are reused in biological cycles or accumulate in the environment. In the presence of oxygen, microorganisms break down materials into carbon dioxide, water, mineral salts, and carbon-rich biomass. Cellulose is an indispensable biodegradable membrane material because of its abundant resources, low cost, and environmental friendliness [5]. Cellulose is a linear polymer of β-(1–4)-D-glucopyranose units, which can be modified by esterification, etherification, oxidation, and reductive amination. After modification, the cellulose derivatives can be used as efficient membrane materials. However, since cellulose and its derivatives are biodegradable, antibacterial modification is highly demanded for the extension of the service life of cellulose-based membranes [5]. Regenerated cellulose, a biopolymer derived from natural sources, is a renewable, biodegradable, and versatile resource. Recycling cellulose from industrial and consumer waste streams presents a valuable opportunity to support the circular economy while mitigating resource scarcity. However, conventional cellulose membranes often suffer from functional limitations, particularly in their susceptibility to microbial fouling. Regenerated cellulose fiber manufacturing processes, like those used to create Viscose/Rayon, use renewable wood pulp cellulose. However, this fiber-spinning process uses highly aggressive chemicals, like carbon disulfide, sulfuric acid, and sodium hydroxide, which leave a large environmental footprint. Recent investigations have proved that regenerated cellulose fiber can be manufactured using environmentally benign solvents.

Recovered polymers can be transformed into functional membranes via solution casting, phase inversion, or electrospinning. Electrospinning, due to its ability to create membranes with nanoscale features, is particularly promising. This section reviews the advantages of electrospun membranes in water filtration, medical devices, and energy storage. Therefore, this research paper proposes a new State-of-the-Art approach to optimize and determine the most suitable process parameters for producing membranes of desired size and properties using mathematical modeling and AI tools in the testing of the systems. After dissolution, process parameters are optimized in the electrospinning of novel membrane prototypes. Electrospinning is a process for preparing a nanofiber from a liquid jet in an electrostatic field. Electrospinning techniques are used to produce continuous nanoparticle fibers of different types of polymers, which allows the obtaining of unique properties of polymer materials intertwined in a high porosity structure. Due to its large specific surface, high porosity, pore connection, ability to control fiber morphology, and ability to spin different materials, electrospinning materials have wide applications in sensors, filtration, protective textiles, catalysts, tissue engineering, and the transfer and release of functional components. The complexity of the process is manifested in a series of parameters that affect the properties of the material, thus opening up a huge number of possible products with new functional properties.

**Figure 2:**
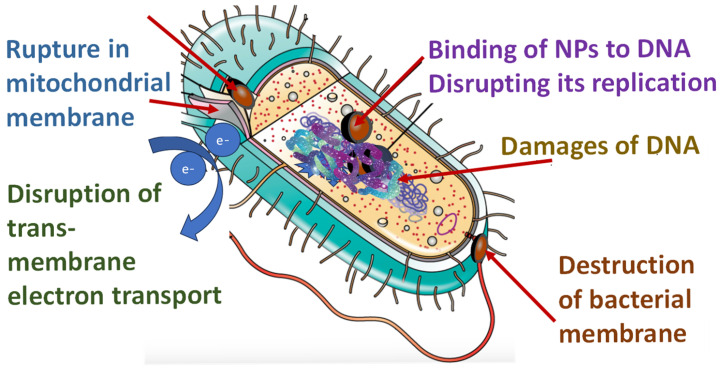
Effects of metal nanoparticles against microorganism *Staphyloccocus mutans* cell due to formation of pits or pores in bacterial cell wall or rupture in mitochondrial membrane [6,7,8].

Membranes for biomedical and pharmaceutical usage need to have desired physical and chemical properties. Nanoscale surface modifications are the current State of the Art in the functionalization of membranes as they reduce and/or inhibit bacterial fouling. Shapes are important factors that affect the biological interaction of microorganisms and membranes, as well as their size, surface area to volume ratio, crystallinity, and surface charge. Antimicrobial effects occur through biochemical reactions, the production of reactive oxygen species (ROS), or ionic release, while modified membranes create a physically hostile surface for bacteria, killing cells via biomechanical damage (Figure 2).

Self-sterilizing antimicrobial biodegradable membranes are designed to continuously inhibit microbial growth and eliminate pathogens upon contact, reducing the need for external disinfection processes. These membranes incorporate antimicrobial agents, such as silver nanoparticles (AgNPs), copper, zinc oxide, or photocatalytic materials, like titanium dioxide (TiO₂), which generate reactive oxygen species (ROS) under light exposure to degrade microbial cells. The self-sterilization mechanism ensures long-term antimicrobial efficacy by preventing biofouling and microbial colonization, making them ideal for applications in water filtration, biomedical devices, and air purification. Additionally, the incorporation of hydrophobic coatings can further prevent bacterial adhesion, enhancing membrane durability and performance. This technology plays a crucial role in maintaining hygiene and safety in critical environments while reducing reliance on chemical disinfectants. Research on antimicrobial membranes was conducted by Li S. et al., who produced PVA/AgNP/BPTA membranes with high antibacterial activity [9]. Electrospun membranes containing Ag nanoparticles (AgNPs) have very good mechanical properties, low water vapor transmittance, and high antibacterial activity [6,9]. In the filtration test, the electrospun nanofibrous membranes show high filtration efficiency. Limaye et al. showed that by increasing the amount of Ag NPs in composite materials, the optical transparency of membranes decreases, while thermal stability is improved for the highest Ag content [10]. Therefore, transforming waste cellulose into efficient composites is a promising way to tackle part of the problems concerning high textile consumption in the EU. Furthermore, Al-Dhabi et al. demonstrated visible antimicrobial activity against pathogenic wound infections such as Bacillus subtilis, Enterococcus faecalis, Staphylococcus epidermidis, multidrug-resistant Staphylococcus aureus, and Escherichia coli [11]. Silver NPs, with their own antimicrobial activity in combination with antibiotics, enhance the action of antibiotics in the treatment of resistant Staphylococcus strains [12].

The use of antimicrobial composite membranes continues to increase year after year, mainly because of their superior properties needed in the pharmaceutical industry and medical engineering. Silver NPs have strong antibacterial activity on a wide range of Gram-positive and Gram-negative bacteria [6,13,14,15]. Sweet et al. have shown that silver NPs have higher antibacterial activity due to their high surface and volume ratio, ensuring better contact with microorganisms [13].

Advanced membranes may consist of many different polymers (Table 1). When two or more layers are combined with antimicrobial agents, novel composites with distinct chemical or physical characteristics are produced, allowing the combination of different properties such as permeability, antimicrobial activity, biocompatibility, strength, and durability. Due to such properties, membranes are useful materials for the pharmaceutical industry in sterilization processes and the preparation of sterile solutions in infusion production (Table 1).

Biocompatibility is critical in drug manufacturing and sterile processes, and when this property is combined with resistance to fouling, excellent applications can be achieved. Membranes used for the filtration of pharmaceuticals are made from a range of polymers selected for their biocompatibility, chemical resistance, mechanical strength, and filtration efficiency, such as cellulose acetate (CA) and regenerated cellulose (RC), which are biodegradable and hydrophilic and are used in sterile filtration and the pre-filtration of sensitive biological compounds. In the simplest case, appropriately adding nanoparticles to a polymer matrix can enhance its performance and reduce biofouling. Surface properties, like hydrophobicity, hydrophilicity, roughness, and charge, are crucial in controlling biofouling on membranes, as they influence membrane–bacteria interactions [16,17,18]. Biofouling is defined as the initial microbial deposition and secondary growth toward forming a biofilm. Diverse microorganisms in the feed solution can deposit on the membrane surface, facilitated via electrostatic interaction between the membrane and bacteria, and then grow and multiply, forming a mixed-culture community called a biofilm. It acts as an extra mass transport barrier and eventually affects membrane integrity and separation process productivity. Considering all fouling types forming on the membrane surface, biofouling is generally accepted as the most complex and challenging phenomenon. This complication originates from the nature of the biofilm community and the ability of microorganisms to reproduce rapidly, whereby high resilience forms against the surrounding environment whenever nutrients are present in the feed solution [19].

In the medical field, polymeric membranes are widely employed as absorbent substrates for biological testing. One prominent application is in diagnostic toolkits utilizing techniques such as dot blotting. This conventional method, which relies on porous membranes as adsorbents, is particularly advantageous due to its simplicity, cost-effectiveness, and ease of use, making it suitable for deployment in regions with limited access to advanced laboratory infrastructure or trained personnel. Based on the antigen–antibody interaction, these assays enable the detection of specific biological targets within human samples. As an early method of qualitative analysis, dot blotting provides rapid and visually interpretable results through straightforward instructions.

However, the use of biological components such as antibodies or antigens is often constrained by their limited availability and the high costs associated with their extraction and purification.

To maximize production efficiency, diagnostic kits are often prepared using minimal concentrations of these biomolecules. Nevertheless, at low protein concentrations, an undesirable ring-like deposition pattern—commonly known as the coffee-ring effect—may form on the membrane surface, where solute particles migrate and accumulate at the edges of the drying droplet.

The coffee-ring effect, a phenomenon where particles accumulate at the edges of a drying droplet, can impact the uniformity of polymeric membrane surfaces during fabrication. This effect often leads to the heterogeneous distribution of fillers or functional additives, which may affect membrane porosity and performance. In membrane-casting processes, controlling solvent evaporation and droplet dynamics is crucial to minimizing this effect. Strategies such as modifying solution viscosity or substrate properties are commonly employed to suppress the coffee-ring effect and achieve more uniform membrane structures.

Shahrudding et al. studied the coffee-ring effect observed on two different polymeric membranes with an understanding of the membranes’ morphology [20]. The evaporation of solution droplets deposited on solid substrates often leads to the formation of ring-like structures, commonly referred to as the “coffee-ring” effect. In this study, nitrocellulose and polyvinylidene fluoride (PVDF) membranes were characterized using water contact angle (WCA) measurements, Fourier-transform infrared spectroscopy (FTIR), scanning electron microscopy (SEM), and atomic force microscopy (AFM), prior to assessing porosity and average pore size. Immunoglobulin G (IgG) from bovine serum was subsequently spotted on the membranes, and the dried spots were stained with Ponceau S dye. The stained membranes were scanned and quantitatively analyzed using Image-J software (1.8.0_172). Distinct ring-like deposition patterns were observed on each membrane type, influenced by the previously characterized surface and structural parameters. PVDF exhibited higher overall porosity but a lower mean pore size and narrower pore size distribution compared to nitrocellulose. Both membranes displayed visible ring-like patterns in side-profile imaging of the protein spots; however, nitrocellulose demonstrated a reduced coffee-ring effect. This is attributed to its more uniform pore size distribution and increased surface roughness, which contribute to lower contact angles and enhanced capillary flow. These properties promote faster solvent evaporation and more homogeneous particle deposition.

Microplastic pollution has emerged as a critical environmental concern, with tiny plastic particles now found in oceans, rivers, soil, and even human tissues. These particles, often originating from consumer products or plastic degradation, pose risks to aquatic life and potentially human health. Conventional water treatment systems are not fully effective in removing microplastics, prompting the need for advanced filtration solutions. Polymeric membranes are being developed to target microplastic particles based on size exclusion and surface interactions, offering promising results in laboratory and pilot-scale studies. To enhance the functionality of these membranes, antimicrobial coatings are being incorporated into their surfaces. These coatings help to prevent biofouling—a major issue in membrane systems—by inhibiting microbial growth and biofilm formation. Materials such as silver nanoparticles, quaternary ammonium compounds, and natural antimicrobial agents have shown effectiveness when integrated into membrane surfaces. By reducing microbial colonization, these coatings improve membrane longevity and maintain filtration efficiency. Moreover, antimicrobial membranes can simultaneously address microplastic contamination and microbial pathogens in water. This dual function is particularly valuable in decentralized or portable water treatment systems. Continued innovation in coating techniques, such as layer-by-layer assembly and surface grafting, enhances coating durability and safety. These developments are paving the way for more sustainable and effective membrane-based solutions to tackle global water quality challenges.

Due to the necessity of thoroughly understanding protein diffusion within polymeric membranes, mathematical models continue to be developed and refined [21]. A common conclusion drawn from these efforts is that diffusion through the membrane typically represents the rate-determining step in the overall transport process. This limitation arises from the physical resistance imposed by the three-dimensional pore structure of the membrane, which forces diffusing molecules to follow elongated, tortuous pathways, thereby affecting the overall mass transfer efficiency. As a result, theoretical modeling must be conducted in parallel with experimental investigations to better elucidate the complex phenomena occurring within the membrane matrix. Ahmad et al. investigated nitrocellulose (NC) membranes, which are widely used for transport media applications related to immunoassays [21], and the effects of membrane pore size on lateral diffusion. They concluded that understanding the diffusion phenomenon would be a useful tool for the design of membrane properties and the customization of specific membrane applications in immunoassays. This is in agreement with the findings of Kalospiros et al., who monitored three simultaneous processes on polymers (diffusion, swelling, and crystallization) [22]. They determined that the sorption of low-molecular-weight solutes into glassy polymers frequently induces swelling of the polymer matrix, which may subsequently trigger crystallization within the polymer phase. As a result, three interrelated kinetic processes—diffusion, swelling, and crystallization—occur concurrently and are intricately coupled. In order to monitor and describe these complex interactions, they proposed a model to incorporate the observed physical phenomena, resulting in quasi-linear hyperbolic equations and enabling accurate simulation of the observed processes.

The aim of this work was to model a complex process of electrospinning in order to determine the optimal process parameters for the production of advanced polymer membranes and investigate their physical and chemical properties. Nano- and micro-fibrous filaments obtained by electrospinning represent a new generation of advanced membranes with improved bioactive properties. This is enabled by their ability to mimic the architecture of native biological tissues. Their combination with bioactive molecules can further improve their properties through antioxidant, anti-inflammatory, and antimicrobial effects. In our previous research [23,24,25], we efficiently added copper, silver, or ZnO NPs on the surface of the polymers and electrospun fibers and performed testing using different methodologies. The electrospinning membrane fabrication approach offers a number of advantages over existing State-of-the-Art techniques such as phase inversion, interfacial polymerization, track-etching, and stretching. Electrospinning is a versatile method that employs high voltage to draw charged polymer solutions into ultrafine fibers, creating nonwoven mats characterized by high porosity and a large surface area. These membranes can be tailored at the nanoscale to meet specific application needs, offering enhanced filtration efficiency and functional versatility and allowing for precise control of fiber morphology and pore-size distribution, with the direct incorporation of functional additives, antimicrobial agents, or nanoparticles into the membrane matrix. Additionally, electrospinning operates at relatively low temperatures, making it suitable for heat-sensitive polymers and bio-based materials. Despite these advantages, electrospun membranes typically exhibit lower mechanical strength than those produced by more traditional methods, and care must be taken to manage solvent use and recovery due to the involvement of volatile organic compounds.

Compared to phase inversion, which is a well-established method of producing mechanically robust membranes with less flexibility in morphology, electrospinning provides significantly higher surface area and functional adaptability. Interfacial polymerization, commonly used for producing thin-film composite membranes in reverse osmosis applications, yields dense and selective layers but lacks surface functionality and is susceptible to biofouling. Track-etching and stretching offer precise control over pore size but result in membranes with lower porosity and limited permeability. Melt spinning and blown film techniques, while suitable for high-throughput production, are generally restricted to thermoplastic polymers and cannot achieve the nanoscale fiber structure enabled by electrospinning.

Therefore, electrospinning distinguishes itself through its high customizability, large surface area, and potential for multifunctional performance, making it particularly attractive for addressing emerging challenges such as microplastic removal, microbial contamination, and the development of responsive or hybrid membrane systems. While conventional fabrication techniques remain dominant in large-scale water treatment due to their durability and cost-effectiveness, electrospun membranes are increasingly finding application in high-value or niche markets. With ongoing advancements in mechanical reinforcement and environmentally friendly processing methods, electrospinning holds strong promise as a next-generation membrane fabrication technology.

This article on net-zero technology provides a comprehensive summary of the current strategies, innovations, and materials aimed at reducing greenhouse gas emissions to achieve climate neutrality. It has a clear focus on circular and energy-efficient solutions while presenting a case study in which it highlights the conversion of textile waste into high-performance membranes, demonstrating how waste can be transformed into valuable products with minimal environmental impact. This example illustrates the role of advanced material design and resource recovery in supporting net-zero goals. In this work, we investigate the electrospinning of polycaprolactone polymers since we expect that electrospinning will ensure the optimal membrane structure.

## 2. Materials and Methods

The testing of membranes followed process optimization of the electrospinning process using a central composite design and response surface methodology (RSM) available in the State-Ease (Minneapolis, MN, USA) program for predicting the optimal process parameters [26,27]. Testing included physical tests (surface tension) combined with thorough chemical characterization by methodologies developed in our research group, including optical microscopy and scanning electron microscopy (SEM) [28,29], FTIR [30,31,32,33], TGA, and DSC.

### 2.1. Sample Preparation

To prepare the polymer solution, 1 g of polycaprolactone (PCL) was dissolved in a solvent mixture consisting of 9 mL of dichloromethane (DCM) and 1 mL of dimethylformamide (DMF). The ratio of the DCM and DMF was chosen based on preliminary tests that were in the range of 7:3, 8:2, and 9:1, showing that 9:1 resulted in the best preliminary prototypes of membranes. The mixture was stirred at room temperature using a magnetic stirrer (VELP, MST Digital Magnetic Stirrer, VELP Scientifica, Usmate, Italy)at a constant speed of 330 revolutions per minute (rpm) for a duration of 1 h. This ensured the complete dissolution of the polymer and the formation of a homogeneous solution suitable for the electrospinning process.

### 2.2. Electrospinning

The electrospinning process was previously developed and described in our previous research papers [24]. The process started with the preparation of the polymer dissolution of polycaprolactone (PCL), sourced from Sigma-Aldrich Chemical Company Ltd. in Dorset, UK, with a viscosity ranging between 1.5 and 2.2 dL g^−1^. The solvents used were dichloromethane (DCM) and dimethylformamide (DMF) obtained from Merck, Darmstadt, Germany. The electrospun nano-nets were generated from the polymer solution using a single-nozzle electrospinning setup on an aluminum plate. The electrospinning process employed a high-voltage power supply system (Genvolt, Shropshire, UK) and a syringe pump from Kd Scientific (Holliston, MA, USA). Both the nozzle and wire collector were situated within a box under continuous airflow to eliminate organic vapor emissions during operation.

**Figure 3:**
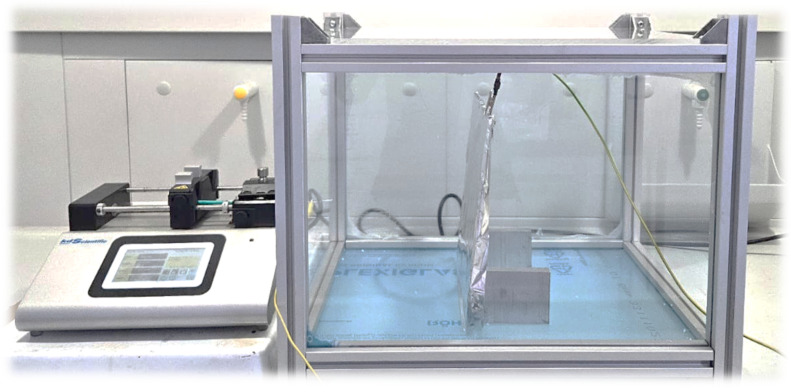
Electrospinning device system, the first prototype built in our laboratory.

The process parameters that were investigated were voltage, the distance between the nozzle and wire collector, and the syringe flow which were tested in our prototype device (Figure 3). Samples after electrospinning were prepared as a potential carrier for drug delivery or biomedical applications and characterized by different sophisticated instrumental methods [34,35,36,37,38,39].

### 2.3. Instrumentation, Protocols, and Data

#### 2.3.1. SEM Investigation of Samples

The surface morphology of the samples was analyzed using a TESCAN VEGA TS5136LS Scanning Electron Microscope (Brno, Czech Republic), equipped with an Energy-Dispersive X-ray Spectroscopy (EDS) detector (Oxford instruments, High Wycombe, UK). SEM-EDS measurements were conducted at a working distance of 25 mm, with an accelerating voltage of 20 kV and magnifications ranging from 1000× to 10,000×. Both backscattered and secondary electron detectors were used, with an image acquisition time of 30 s. Prior to analysis, the samples were coated with a thin Au/Pd layer using a SC 7620 Sputter Coater (Quorum Technologies, Laughton, UK) under the following conditions: 230 V power supply (12 A), a target distance of 45 mm, an output voltage of 800 V, a sputtering rate of 6 nm/min, a coating thickness between 1 and 20 nm, and coating uniformity greater than 10%. Argon was used as the sputtering gas at a pump rate of 25 L/min.

#### 2.3.2. FTIR Investigation

Fourier-transform infrared and Raman spectroscopy were performed using a BRUKER EQUINOX 55 FT-IR/FT-Raman spectrometer (Billerica, MA, USA), equipped with an FRA 106/S Raman module and an Nd:YAG laser (1064 nm) (Billerica, MA, USA). The spectral range for IR analysis was 15,000–4000 cm⁻^1^ (NIR) and 4000–400 cm⁻^1^ (MIR), while Raman spectra were collected in the 3500–400 cm⁻^1^ range. The system included sample holders and cells for solid, liquid, and gas phases, as well as accessories for temperature-controlled measurements, attenuated total reflectance (ATR), diffuse reflectance (DRIFT), and in situ analysis via fiber optic probes.

#### 2.3.3. TGA Analysis

Thermogravimetric analysis (TGA) was conducted to determine the thermal degradation temperatures of the polymeric mixtures and experimentally quantify the indomethacin content incorporated within the polymer blends. The analysis was performed using a Mettler Toledo TGA/DSC 3+ instrument (Mettler Toledo, Zagreb, Croatia) under a programmed heating protocol ranging from 35 °C to 600 °C. The heating rate was set at 10 °C per minute, and the measurements were carried out under a controlled nitrogen atmosphere with a constant flow rate of 50 mL per minute. High-purity nitrogen gas (UTP grade, purity 99.999%) was used to prevent oxidative degradation during thermal treatment. Samples were placed in 150 µL platinum crucibles, and the sample mass ranged between 10 and 15 mg.

### 2.4. Modeling and Optimization of Process Parameters for Effective Electrospinning

Statistical analysis was performed using Design Expert Stat Ease software 9.1 (Minneapolis, MN, USA). The central composite design (CCD) was chosen as it is especially useful in studies where the goal is to optimize a process or system because it is tailored to accurately model second-order (quadratic) relationships. These quadratic models include not only linear and interaction terms but also squared terms, which are essential for capturing curvature in the response surface. More detailed responses often do not change linearly with input variables, and simple linear models are insufficient for predicting optimal conditions. The CCD addresses this by including axial (star) points, which extend beyond the typical factorial design space and allow for the detection of curvature. In contrast, a full factorial design at three levels for each factor would be required to model this same curvature, and this dramatically increases the number of experimental runs, especially as the number of variables grows. The CCD uses a strategic combination of factorial, axial, and center points to provide the same level of information with fewer experiments, making it a more efficient and cost-effective approach for fitting second-order models.

A central composite design was chosen based on our previous experience [26,27] of the modeling of complex systems with more than three process parameters with replicates, so the total number of experiments was 20.

The optimal conditions were calculated using algorithms within the program of Design of Experiments (DoEs), which is designed to systematically plan, conduct, analyze, and interpret controlled tests to evaluate the factors that may influence a particular outcome. It enables researchers and engineers to develop predictive models, optimize processes, and identify key variables that affect system performance.

In this case, the optimization process was conducted using the State Ease v.9.1 software, a powerful tool for creating experimental designs and analyzing responses through statistical modeling. The steps followed during the experiment are outlined below:

Steps of Optimization:

(a) Conduct 20 Preliminary Experiments (1–20)

The first step involved designing and performing 20 initial experiments, each varying the levels of three selected input parameters. These experiments were constructed to systematically explore the experimental space and gather data on system behavior;

(b) Develop a Model Describing the System with 3 Input and 2 Output Parameters

Using the results of the 20 trials, a statistical model was developed to describe the relationships between the three input variables (e.g., temperature, concentration, and time) and two output responses (e.g., membrane density and surface area). The model typically takes the form of a polynomial equation derived through regression analysis;

(c) Verify the Accuracy of the Model with New Experiments

To ensure the reliability of the developed model, validation experiments were conducted using new parameter combinations not included in the original 20. The outcomes of these experiments were compared to the model’s predictions to evaluate its predictive accuracy;

(d) Use the Validated Model to Predict Desired Optimal Values

Once the model was confirmed as accurate, it was used to predict optimal values of the input parameters that would result in the maximum density with minimum surface area—a desirable outcome for specific membrane applications. Graphical tools such as response surface plots and contour diagrams were used to identify these optimal conditions within the design space.

Through these steps, the DoE approach allowed for a quantitative understanding and optimization of the system, providing both efficiency and robustness in experimental research. By reducing the number of experiments needed and revealing key interactions between variables, the DoE approach proves essential in fields like material science, chemical engineering, and advanced manufacturing.

### 2.5. Droplet Test

The droplet test is a simple yet informative method used to evaluate the surface absorption behavior of membranes, particularly in relation to wettability and liquid transport properties. In this procedure, a controlled volume of liquid—typically 1 μL of a sample—was carefully placed on the membrane surface using a precision syringe or micropipette equipped with a standardized needle. The membrane was then observed under ambient conditions, and the time it took for the droplet to be fully absorbed into the membrane was recorded in seconds. This absorption time reflects key surface characteristics such as porosity, surface energy, and fiber packing density. A shorter absorption time indicates better wettability and higher capillary action, which are often associated with uniform pore distribution and higher membrane density. The droplet test is especially useful in studies investigating the coffee-ring effect, as it provides insight into how liquid behavior at the microscale interacts with the membrane structure to influence the spread and distribution of solutes.

## 3. Results and Discussion

### 3.1. Modeling of the Electrospinning Process for Producing Biodegradable Membrane

Before modeling the electrospinning process for producing biodegradable membranes, we tested many materials and varied experimental conditions and amounts of solvents and polymers in the preliminary experiments in order to obtain a control sample and establish the required ranges of the working parameters. The system for electrospinning is the first prototype device that we are building in our laboratory, and in this first step, it has only one target Al plate and no built-in sensors. In our next experiments, we plan to apply different movable targets and incorporate temperature and humidity sensors that will record data automatically during the electrospinning process.

The choice of a reliable polycaprolactone (PCL) polymer for efficient electrospinning is also very important. It has been consistently demonstrated that using polymers with average molecular weights of 45,000 or 80,000 g/mol, typically at concentrations between 5 and 20 wt%, efficient membranes can be demonstrated. In contrast, the literature data show that there are successful reports on electrospinning of low-molecular-weight PCL (Mw = 14,000 g/mol). Our unsuccessful experiments also proved this theory. The inability to form uniform fibers at lower molecular weights is often evident from the appearance of beads along the fiber, a phenomenon attributed to insufficient surface tension or inadequate molecular weight, both of which impair the formation of a stable and continuous polymer jet. Specifically, lower molecular weights lead to reduced polymer chain entanglement, a critical factor in fiber formation, even when higher concentrations are used. As a result, instead of forming fibers, these solutions frequently undergo electrospraying, yielding microspheres. Indeed, low-molecular-weight PCL has been shown to produce microspheres across a broad concentration range, up to 30% *w/v* (21.4 mM) [40]. Therefore, in all our experiments, a high-density PCL was applied.

Optimizing for a smaller membrane diameter plays a crucial role in enhancing membrane performance. A reduced diameter typically leads to a more uniform and controlled membrane thickness, which is essential for achieving consistent structural integrity. Thinner, well-distributed membranes often exhibit improved barrier properties, as they reduce the likelihood of defects or inhomogeneities that could compromise selectivity. Additionally, a smaller diameter can enhance the mechanical strength and handling properties of the membrane, which are important for practical applications in filtration or separation technologies.

The installation of sensors for pressure and temperature, as well as additional components for sample collection—such as cylinders and movable lateral elements—is currently underway. These upgrades are essential for advancing the experimental setup and enabling more robust process control and data acquisition. Once this integration is complete, the Technology Readiness Level (TRL) of the system can be elevated from a laboratory-scale configuration to a pilot-industrial level, marking a significant step toward practical application and industrial implementation.

This study represents only the initial set of experiments aimed at optimizing the experimental setup for membrane fabrication. The primary focus was to establish reliable control over physical parameters critical to the development of the laboratory-scale system. Future work will expand upon these findings by investigating microbial resistance and potential self-sterilization properties of the membranes. However, at this stage, such claims remain speculative without thorough in vitro and in vivo validation. Additionally, future studies will include comparisons with benchmark membranes—either commercial products or those reported in prior research—to provide a more robust evaluation of performance and functionality.

#### Modeling of the Membrane Height

Modeling was conducted through three phases:

- The model was proposed;
- The model was evaluated;
- The optimal combination of parameters was predicted.

A total of 22 electrospinning experiments were conducted, with each experiment lasting 15 min (20 were used for modeling and 2 for the verification of the adequacy of the obtained model). The verification experiments were performed in triplicate *(n* = 3) in order to verify the accuracy of the methodology. Moreover, the measured temperature in the laboratory was 24 °C, and the humidity was 60%. As a control group, we tested PDA electrospun materials obtained from a collaborating laboratory. However, as this is the first prototype of the electrospinning system that we are building in our laboratory (Figure 3), the process of incorporating working sensors within the device in order to monitor the changes in the process parameters (such as temperature or humidity), as well as for solvent recovery, is still under construction.

After the electrospinning process, the dimensions of the resulting fiber mats—specifically, their height and width—were measured in order to evaluate the consistency and uniformity of the deposited membranes. Additionally, a droplet absorption test was performed to assess the membrane’s wettability. In this test, a single droplet of water with a volume of 1 microliter (μL) was gently placed on the surface of each membrane, and the time required for complete absorption of the droplet was recorded in seconds. This time serves as an indicator of surface porosity and absorption efficiency. During electrospinning, fibers were dispensed through a needle with a gauge size of 22, which directly influences fiber thickness and deposition. The measured data, including membrane dimensions and droplet absorption times, are systematically presented in Table 2 for further analysis and comparison.

The droplet test provided valuable insights into the suppression of the coffee-ring effect in relation to the membrane-surface properties. Specifically, it was observed that membranes with larger surface areas tend to exhibit a lower density, a factor that directly contributes to the formation and intensity of the coffee-ring effect. Lower-density membranes enable more pronounced particle migration toward the droplet’s periphery during evaporation, thereby enhancing the ring formation. Conversely, denser and more compact membrane structures facilitate uniform absorption, thereby mitigating this effect.

Our experimental results support this relationship. The shortest recorded absorption time—10.4 s—was observed in Experiment 10, which was conducted under the lowest applied voltage and with corresponding parameter values of 10.3 and 13.136. This indicates more efficient droplet absorption and better suppression of the coffee-ring effect. In contrast, the longest absorption time was observed in Experiment 1, which was performed at the highest voltage setting, with parameter values of 10.57 and 12.835. These conditions likely resulted in a more open and less uniform fiber structure, contributing to a slower absorption rate and a more prominent ring-like pattern. The correlation between voltage, membrane density, and droplet behavior highlights the importance of electrospinning parameters in controlling surface morphology and improving the performance of membranes for diagnostic applications. For validation of the robustness, some measurements were performed in triplicate (*n* = 3).

Surface height and surface width of the membrane were selected as key parameters for optimization because they directly support the development and fine-tuning of the laboratory apparatus, which we are building and optimizing from scratch. These surface characteristics provide essential feedback for adjusting process conditions and ensuring consistency in the membrane formation. In particular, the more complex model obtained for surface width highlighted the sensitivity of this parameter to external influences such as airflow. This insight demonstrated the need for implementing a housing structure around the apparatus to shield it from ventilation effects within the laboratory hood, which were found to interfere with the reproducibility and accuracy of the results.

The first model was obtained for diameter height using State Ease software based on Table 2 as:

Diameter width = + 6.31755 − 0.29903 × Power + 0.43543 × Distance − 0.047677 × Flow(1)

The statistical analysis of the model was performed using ANOVA. It reveals that the regression model was statistically significant. The model F-value was 5.81, and the corresponding *p*-value was 0.0070, indicating that the variation explained by the model was unlikely due to chance, with only a 0.70% probability that such a result could have occurred as a result of random noise. This established a strong basis for the model’s relevance in interpreting the relationship between the variables and the response.

Among the three independent variables analyzed, power (factor A) and distance (factor B) were found to be significant contributors to the model. Power had a *p*-value of 0.0085, while distance had a *p*-value of 0.0245, confirming that changes in these factors had a meaningful impact on the response variable. On the other hand, speed flow (factor C) showed a *p*-value of 0.7653, indicating that it did not significantly affect the response under the tested conditions. This suggests that speed flow might be excluded from the model in subsequent refinement, provided that its removal does not violate the model hierarchy.

In the context of the Design of Experiments (DoEs), an equation expressed in terms of actual factors was used to predict the response variable at specific, real-world values of each experimental factor. These values must be entered in their original measurement units (e.g., temperature in °C, pressure in bar, or concentration in mg/mL), not as coded values. While this predictive model is useful for estimating outcomes under various process conditions, it is important to note that it should not be interpreted to assess the relative importance or contribution of each factor. This is because the regression coefficients in the equation are scaled according to the units of the factors, and the intercept does not represent the average response but rather the response at the zero level of all factors, which may lie outside the experimental region.

**Figure 4:**
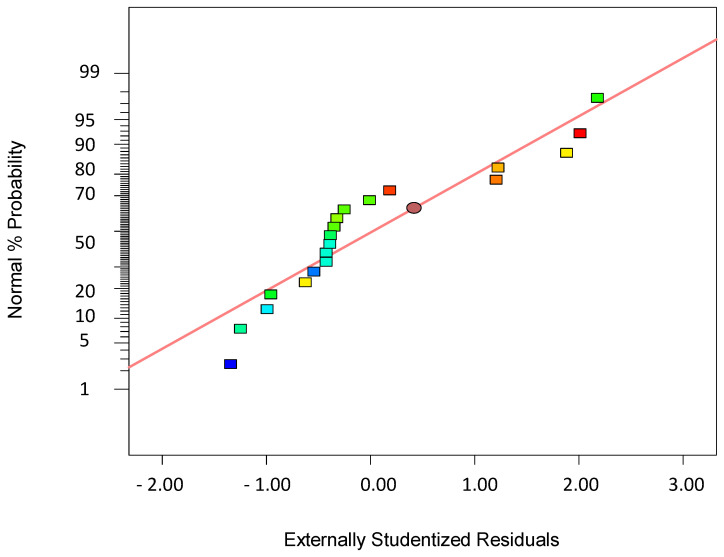
Normal plot of residuals for the first model (1).

**Figure 5:**
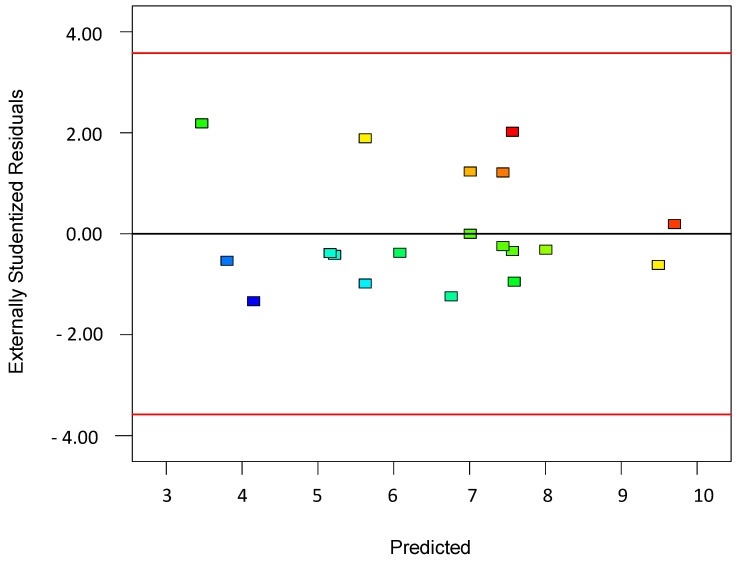
Plot showing residuals versus predicted values for the first model.

**Figure 6:**
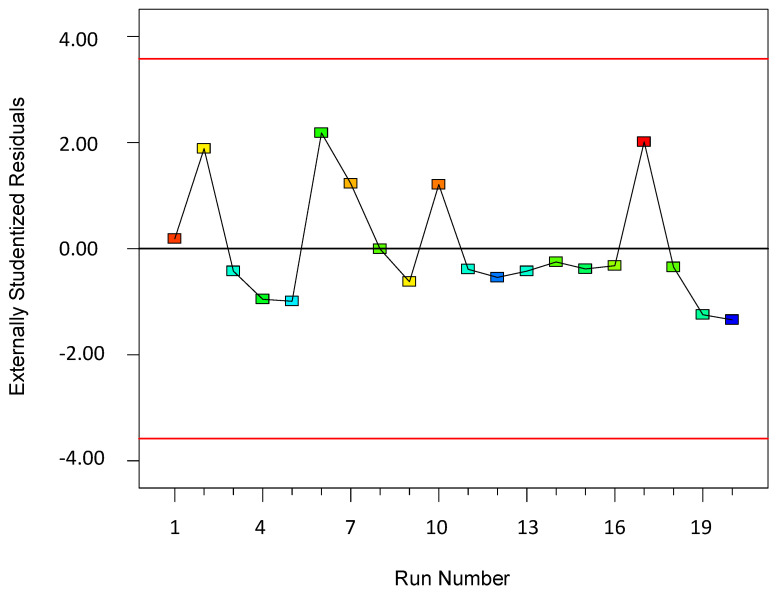
Plot: residuals vs. run for the first model (1).

**Figure 7:**
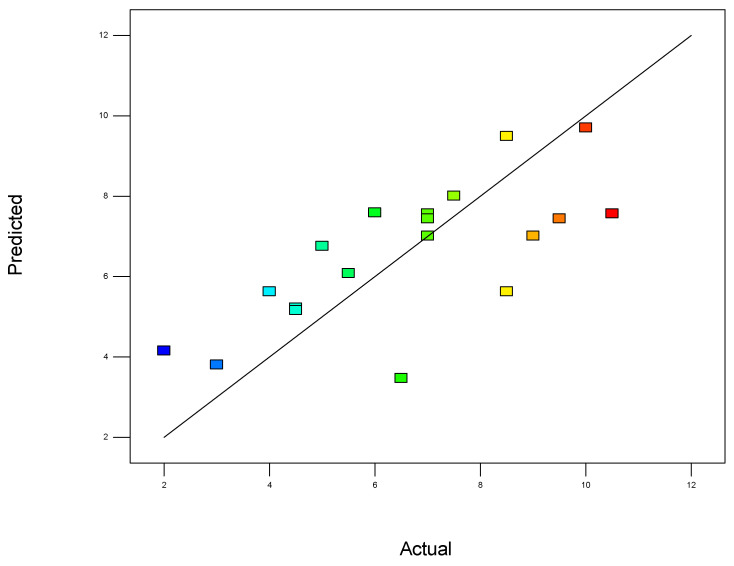
Predicted values vs. actual values for the first model (1).

**Figure 8:**
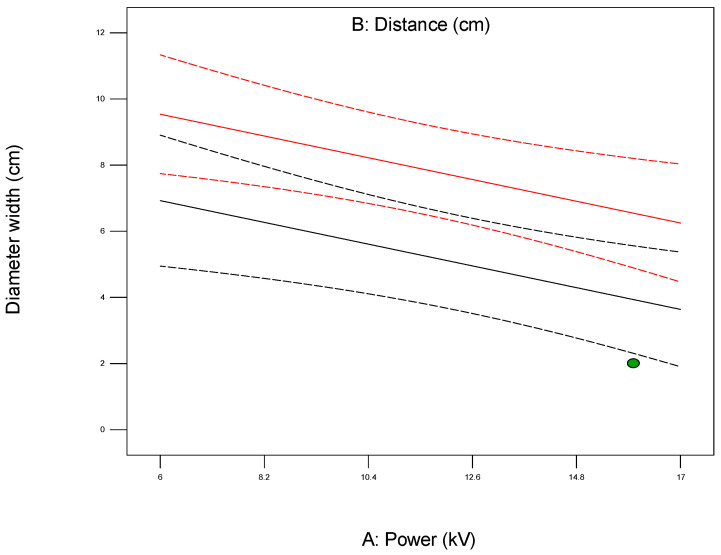
Plot showing interaction between parameters being investigated for the first model (1).

**Figure 9:**
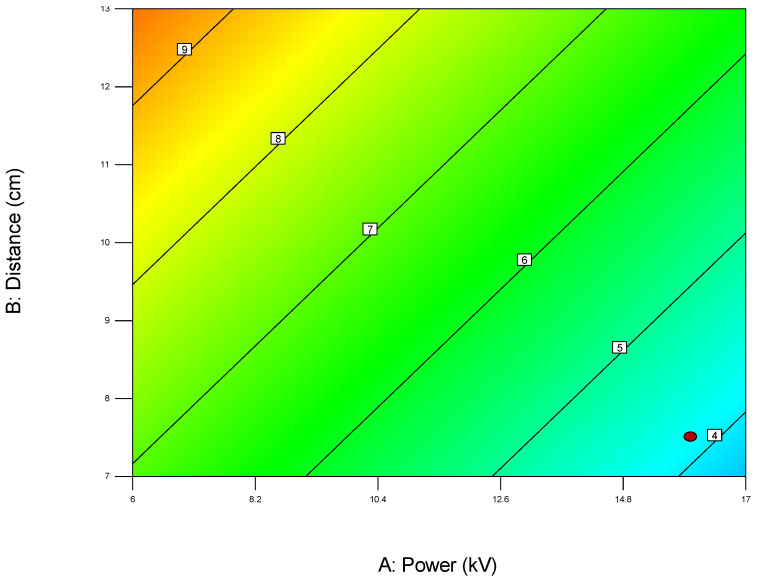
Two-dimensional contour plot with response values on the two investigated parameters A (power) and B (distance) on the diameter width of the membrane (red area—maximal output, blue area—minimal values, red dot presents one of the most favorable predicted optimal solution) obtained by using the first model (1).

**Figure 10:**
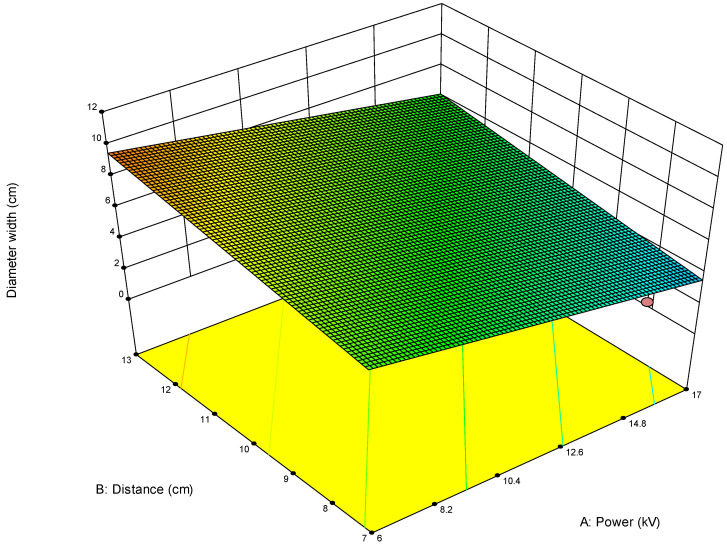
Three-dimensional contour plot showing dependance of response parameter on parameters A (power) and B (distance) on the diameter width of the membrane obtained for the first model (1). In this Figure the red circle under the blue area presents the predicted optimal value for the first parameter within the model (1).

**Figure 11:**
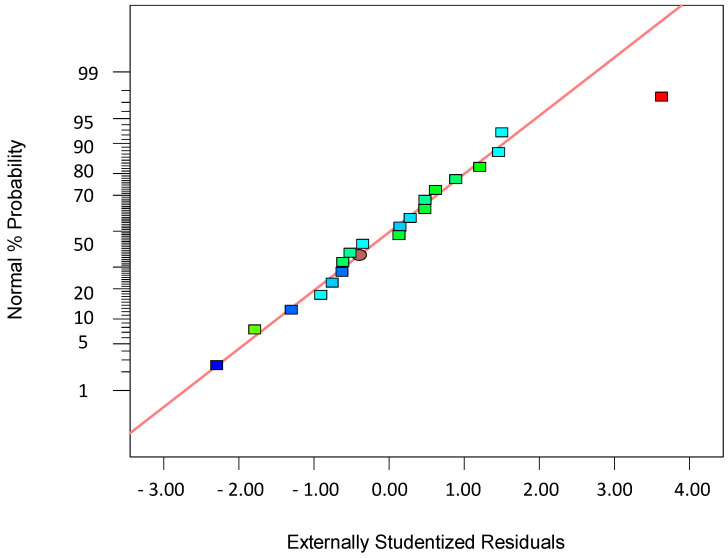
Normal plot of residuals obtained for the second model (2).

**Figure 12:**
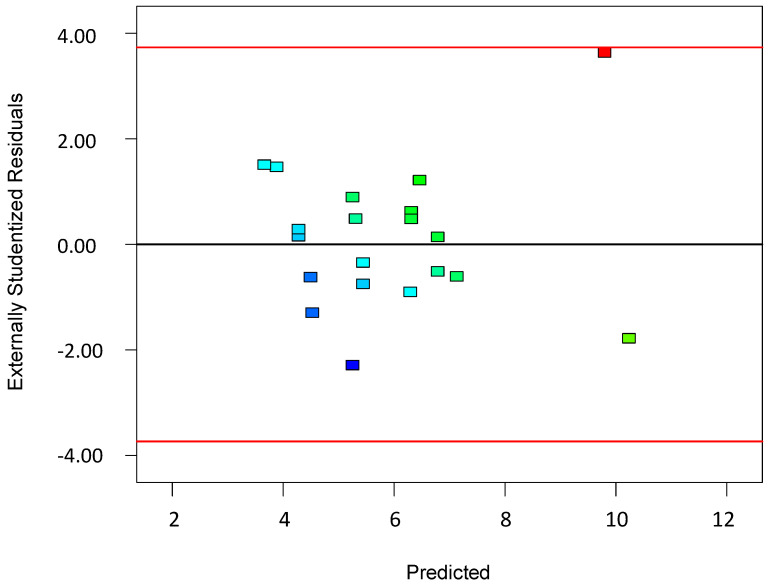
Plot: residuals vs. predicted obtained for the second model (2).

**Figure 13:**
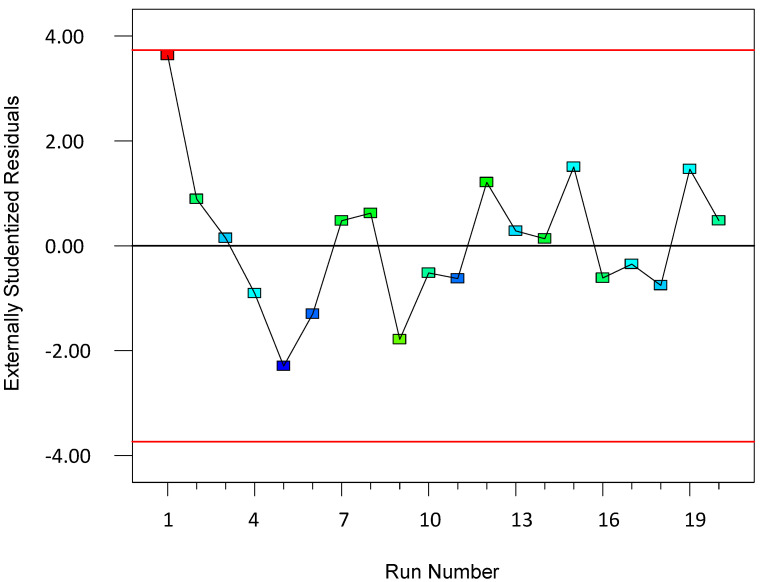
Plot: residuals vs. run obtained for the second model (2).

**Figure 14:**
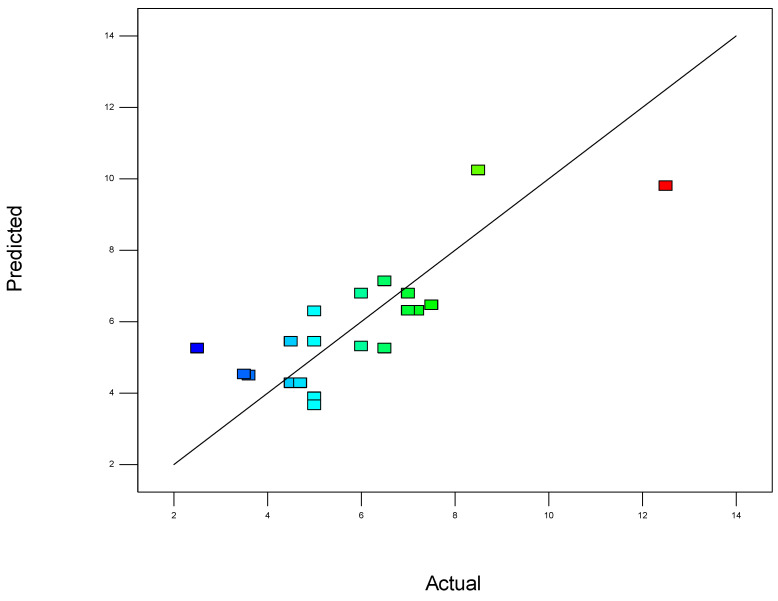
Plot: predicted vs. actual obtained for the second model (2).

**Figure 15:**
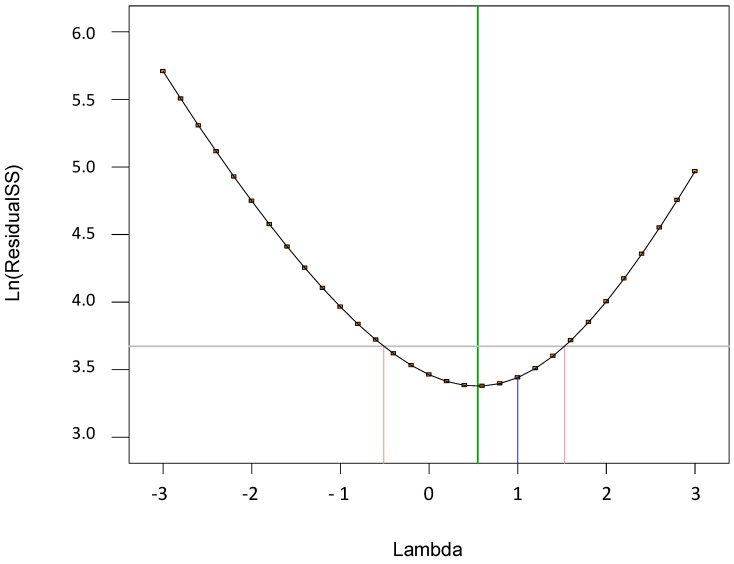
Plot: Box–Cox for power transformation obtained for the second model (2).

**Figure 16:**
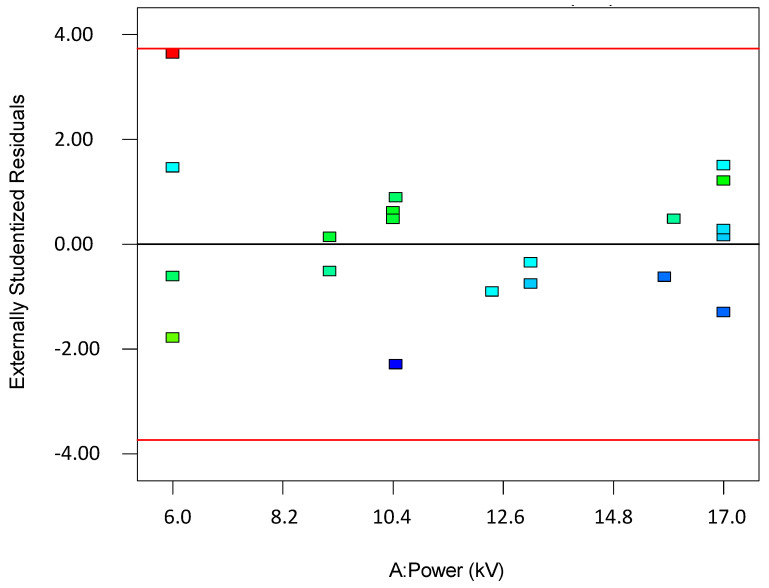
Plot: residuals vs. actual obtained for the second model (2).

**Figure 17:**
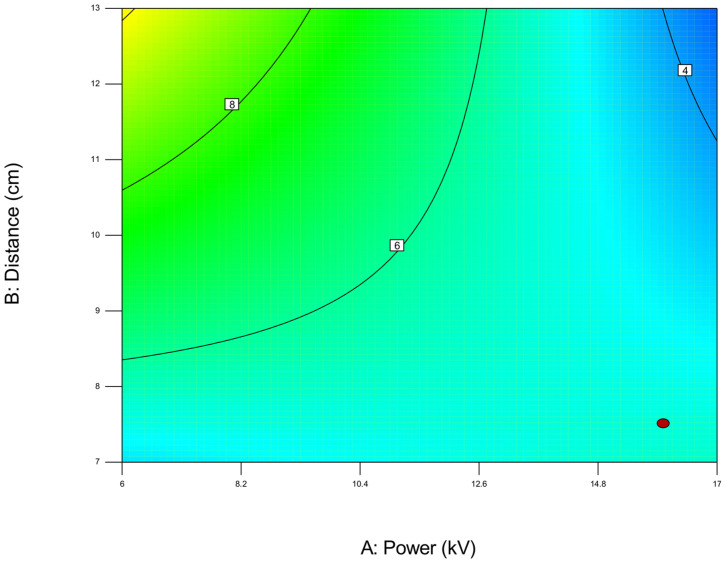
Two-dimensional contour plot of the influence of parameters A (power) and B (distance) on the diameter height of the membrane obtained for the second model (2), and compared to the predicted optimal value presented as a red dot.

**Figure 18:**
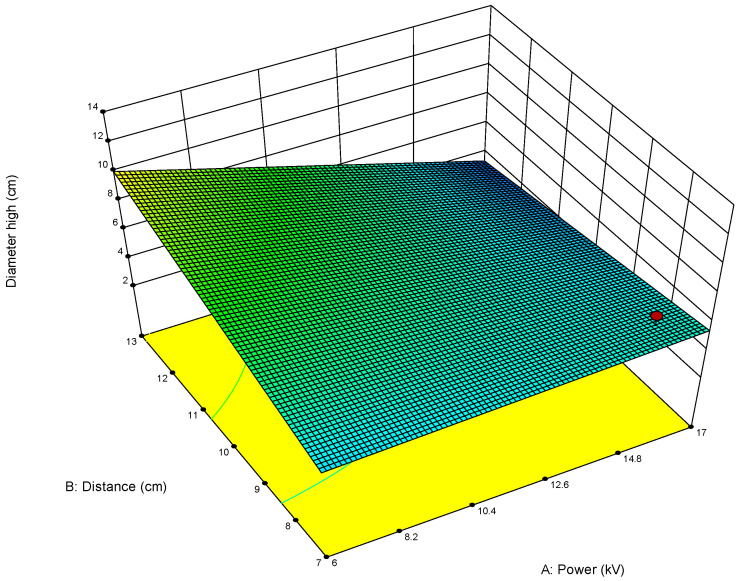
Three-dimensional contour plot for optimization of the second diameter on parameters A (power) and B (distance) on the diameter height of the membrane obtained for the second model (2), predicted optimal minimal value is graphically presented as a red dot in the most favorable area that was further tested by experiments.

**Figure 19:**
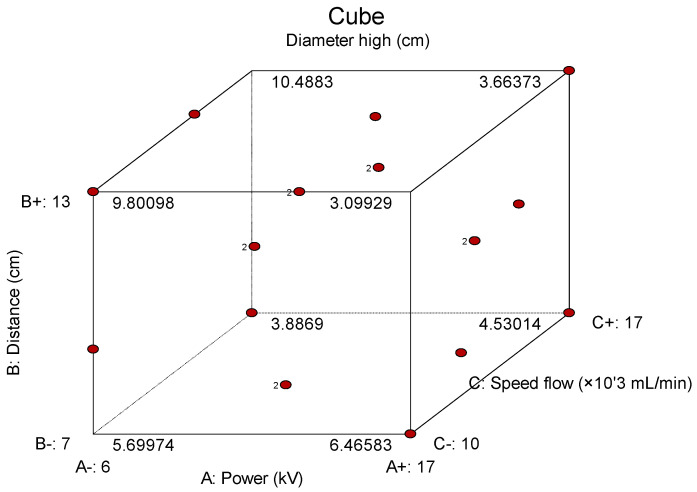
Cube plot with experimental points where parameters investigated are A (power), B (distance) and C (speed flow).

**Figure 20:**
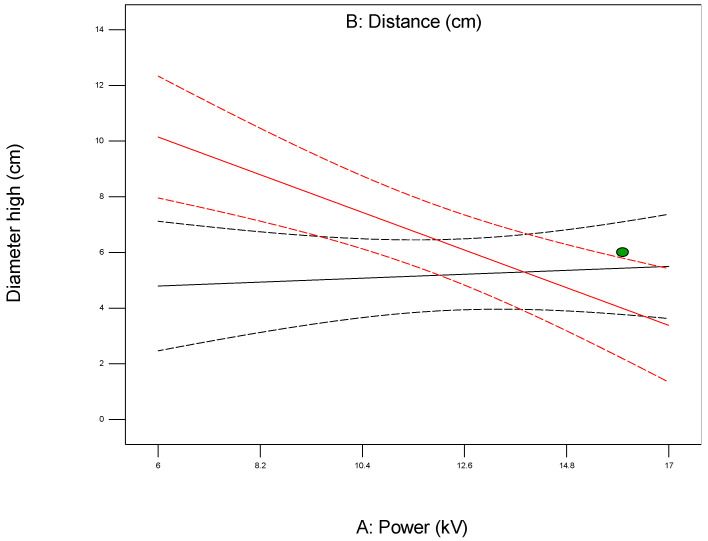
Interaction plot.

To validate the quality of the model, it is essential to proceed with diagnostic plots (Figure 4, Figure 5, Figure 6, Figure 7, Figure 8, Figure 9 and Figure 10 for the first parameter of the optimization, sample height, and Figure 11, Figure 12, Figure 13, Figure 14, Figure 15, Figure 16, Figure 17, Figure 18, Figure 19 and Figure 20 for the second output parameter, sample width). Firstly, the normal probability plot of the studentized residuals is examined to ensure that the residuals (the differences between the observed and predicted values) follow a normal distribution, indicating a good model fit. Second, the plot of the studentized residuals versus the predicted values helps assess whether the variance of residuals is constant across the range of predictions, confirming the assumption of homoscedasticity. Third, the plot of the externally studentized residuals is used to detect outliers or influential data points that may unduly affect the model’s accuracy. Lastly, the Box–Cox plot helps determine whether a transformation of the response variable is necessary to meet model assumptions, such as normality and constant variance. If all diagnostic statistics and plots indicate a robust model, the process can be finalized by generating and interpreting the model graphs, which visually represent the influence of factors on the response and assist in identifying optimal operating conditions. Moreover, the model was verified through a comparison of the predicted and obtained data (Table 3), which showed errors in the range from 2% (which is excellent) to 16% (which is average).

After successful modeling, the model can be applied for optimization of the desired system parameters for maximal or minimal density of the material, maximal or minimal surface tension, or any other desired property (Table 4). Our goal was to minimize the surface and maximize the density of the nanofibers in the material.

Figure 6, Figure 7, Figure 8, Figure 9, Figure 10, Figure 11, Figure 12, Figure 13, Figure 14, Figure 15, Figure 16, Figure 17, Figure 18, Figure 19 and Figure 20 show diagnostic plots for the first and second output parameters (namely, sample height and sample width). Contour plots serve as a valuable tool for visualizing the response surface and validating the accuracy of the fitted model. By illustrating the relationship between independent variables and the response within the experimental domain, these plots allow for the identification of trends, interactions, and optimal regions. When the contour plots align with the predicted behavior of the model and show smooth, consistent transitions, they support the model’s adequacy in describing the system. Furthermore, the shape and orientation of the contours can indicate the presence of significant variable interactions and help confirm whether the quadratic or interaction terms in the model are meaningful. Thus, contour plots not only aid in understanding the effects of the parameters but also serve as a visual confirmation of the model’s validity and reliability.

The normal probability plot of residuals shown in Figure 4 is a critical diagnostic tool in regression analysis and Design of Experiments (DoEs) used to assess whether the residuals—or errors between the observed and predicted values—follow a normal distribution, which is a fundamental assumption of many statistical models. In this plot, the studentized residuals are plotted against a theoretical normal distribution in such a way that if the residuals are normally distributed, the points will lie approximately along a straight diagonal line. Significant deviations from this line suggest departures from normality, indicating potential problems in the model, such as non-linearity, omitted variables, or incorrect functional form. The residuals are often “studentized”, meaning they are standardized by an estimate of their standard deviation to give a better understanding of their behavior across different levels of prediction. A symmetric spread of the points around the line suggests that the errors are randomly distributed, with no skewness, while systematic curvature or S-shaped patterns may indicate that the data are skewed or that there are heavier tails than the normal distribution predicts. Identifying such issues is essential because non-normal residuals can affect the validity of the hypothesis tests and confidence intervals derived from the model. In practice, the normal plot is often used in conjunction with other diagnostic plots to give a fuller picture of model adequacy. However, mild departures from normality are often tolerable, especially in large sample sizes, due to the Central Limit Theorem. Nevertheless, the normal probability plot remains a simple yet powerful visual tool to initially check one of the key assumptions of linear modeling and guide further investigation if needed.

The *residuals* vs. *predicted values* plot shown in Figure 5 is another essential diagnostic tool in regression analysis and experimental design used to evaluate the assumption of homoscedasticity as the constant variance of the residuals across all levels of the predicted response. In this plot, the residuals (i.e., the difference between the observed and predicted values) are plotted on the y-axis, while the predicted values from the model are plotted on the x-axis. Ideally, the residuals should be randomly scattered around the horizontal line at zero, with no obvious pattern, funneling, or curvature. This would indicate that the variance of the errors is constant and the model is a good fit across the entire range of predicted values.

If the plot shows a fanning out (or in) of residuals, where the spread increases or decreases with the predicted values, it suggests heteroscedasticity, meaning the error variance is not constant, which can violate key assumptions of the regression and lead to inefficient estimates or misleading inference. If the residuals show a systematic pattern such as curvature, it might indicate that the model is missing an important term, like a quadratic or interaction effect. Additionally, the clustering of residuals might point to outliers or incorrect grouping in the data. This plot is particularly useful when evaluating the performance of models created via the Design of Experiments (DoEs), as it provides a visual cue for identifying model inadequacies, outliers, or the need for transformation. It is often examined together with other diagnostics, such as the normal plot of residuals and the Box–Cox plot to ensure robust model interpretation.

The residuals vs. run plot is a diagnostic tool used to detect any trends, drifts, or non-random patterns in the residuals over the sequence of experimental runs. In this plot, the residuals (differences between the observed and predicted values) are plotted on the y-axis, and the run number—the order in which the experiments were performed—is plotted on the x-axis. The key goal of this plot is to assess whether the residuals are randomly distributed across time or experimental order, which is essential for validating the assumption of the independence of errors.

A random scatter of points above and below the zero line, with no discernible trend, a wave-like pattern, or clustering, indicates that the assumption of independence holds true and that the model predictions are stable throughout the experimental period. On the other hand, a systematic pattern—such as a consistent rise or fall in residuals or cyclical behavior—may suggest the presence of time-related effects, instrumental drift, operator influence, or other uncontrolled variables that change over the course of the experiment. These effects could bias the results and weaken the reliability of the model.

In the context of experimental design and statistical modeling, examining the residuals vs. run plot is crucial for ensuring the quality of data and the robustness of conclusions. If non-random patterns are observed, it may be necessary to introduce blocking factors, re-randomize the experiment, or investigate external sources of variation to correct the issue.

The predicted vs. actual plot (Figure 7) is a crucial diagnostic tool used to assess the accuracy and performance of a statistical or mathematical model. In this plot, the predicted values generated by the model are plotted on the x-axis, while the actual experimental or observed values are plotted on the y-axis. Ideally, if the model perfectly predicts the outcomes, all points will lie exactly on the 45-degree diagonal line (the line of perfect agreement). The closer the data points are to this diagonal line, the better the model’s predictions align with the observed results.

This plot provides a clear visual representation of how well the model fits the data. A strong linear trend, where points are tightly clustered around the diagonal, indicates a high degree of accuracy and a good model fit. In contrast, significant deviations from the diagonal suggest biases or inconsistencies in the model, which may indicate underfitting (when the model is too simple) or overfitting (when the model captures noise rather than the underlying trend).

In addition, the predicted vs. actual plot helps to detect any systematic errors, such as regions where the model consistently overestimates or underestimates the response variable. If the spread of points increases or decreases along the diagonal, it may suggest issues like heteroscedasticity, where the variability of errors changes across the range of predictions. Overall, this plot is a simple yet powerful way to visually validate the effectiveness of the model and is commonly used in conjunction with other residual plots and statistical metrics to ensure the robustness and reliability of the conclusions drawn from the experimental data.

The interaction plot shown in Figure 8 is a graphical tool used in the analysis of the Design of Experiments (DoE) to visualize how the levels of two or more independent variables (factors) interact and influence the response variable. Unlike the main effect plots that show the isolated impact of each factor, interaction plots reveal whether the effect of one factor on the response depends on the level of another factor.

In a typical two-factor interaction plot, the x-axis represents the levels of one factor, while separate lines are plotted for the different levels of the second factor. The response variable is shown on the y-axis. If the lines in the plot are parallel, this indicates there is no interaction between the two factors, meaning their effects are additive and independent. However, if the lines cross or diverge/converge significantly, this suggests a strong interaction, implying that the effect of one factor changes depending on the level of the other factor.

For example, when optimizing electrospun membrane properties, factors such as voltage and polymer concentration might interact. An interaction plot helps determine whether increasing voltage consistently increases fiber diameter or if that effect depends on the polymer concentration being high or low. These plots are especially valuable in identifying synergistic or antagonistic effects between variables and are essential in model interpretation and process optimization. A proper understanding of factor interactions leads to better process control and improved performance in applications like material synthesis, drug delivery, or membrane fabrication. The *plot of modeled parameters* is a graphical representation of how the response variable(s) change based on the variation and interaction of input factors in a statistically modeled experimental setup. These plots help in visualizing the predictive model developed through DoE software (such as Design-Expert, v. 9.1.) and provide crucial insights into process behavior, optimization potential, and relationships among variables. In the context of a modeled system, such as optimizing electrospun membrane characteristics, the DoE plots commonly include contour plots, 3D surface plots, and interaction plots, like the 2D plot shown in Figure 9, which uses contour lines to show response values across two factors, keeping others constant. They are particularly useful to identify “sweet spots” where optimal conditions lie. In contrast, the 3D surface plot presented in Figure 10 demonstrates the interaction between two factors and their effect on the response. The colors of the plot represent the minimal and maximal values (in curved areas, peaks, and valleys on the plot).

When used collectively, all of the above-described DoE model plots allow researchers to visualize how changes in factors influence the response, detect non-linear behaviors and interactions, and pinpoint optimal conditions for desired performance, as well as validate model accuracy and reliability through diagnostic plots. In summary, DoE plots of modeled parameters transform complex statistical data into intuitive visual tools, aiding in precise decision-making and robust process development.

### 3.2. Optimization of the Second Parameter

Optimization of the second parameter was performed in the same manner, including modeling with the following steps:

- The model was proposed;
- The model was evaluated;
- Prediction of the optimal parameters.

Model 2: Diameter high = 2.40580 + 0.87764 × Power + 0.76716 × Distance − 0.66609 × Flow − 0.11315 × Power × Distance − 1.59553 × 10^−3^ × Power × Flow + 0.059527 × Distance × Flow (2)

The statistical evaluation of the 2FI (two-factor interaction) response surface model using ANOVA (Type III Partial Sum of Squares) showed that the model was statistically significant. The model F-value was 4.01, with a corresponding *p*-value of 0.0172, indicating that there was only a 1.72% probability that such a large F-value could have occurred due to random variation. This supported the conclusion that the model, as a whole, successfully explained a significant portion of the variability in the response.

Among the factors analyzed, power (A) and the interaction term AB (power × distance) were statistically significant, with *p*-values of 0.0079 and 0.0095, respectively. These values indicated that both the main effect of power and its interaction with distance had a meaningful influence on the response variable. In contrast, the other main effects—distance (B) and speed flow (C)—along with the interactions AC and BC were not significant, as their *p*-values exceeded the 0.05 threshold. In particular, speed flow had a very high *p*-value of 0.5372, while AC showed a nearly negligible F-value, suggesting no interaction effect. The lack of significance in these terms implies that they contributed little to the model and could potentially be excluded in further model refinement, provided that the model hierarchy is maintained.

The model had a standard deviation of 1.55 and a coefficient of variation (C.V.) of 26.26%, suggesting moderate variability in the experimental data. The coefficient of determination (R^2^) was 0.6490, indicating that the model explained about 64.9% of the variation in the response. However, the adjusted R^2^ was lower at 0.4870, reflecting a reduction in explained variability when accounting for the number of predictors. The predicted R^2^ was −0.5063, a negative value indicating that the model had poor predictive ability for unseen data and that using the mean response as a predictor might have yielded better accuracy. Despite this, the Adequate Precision value was 7.174, well above the threshold of 4, suggesting a sufficient signal-to-noise ratio and indicating that the model could still be used to explore the design space reliably.

The lack-of-fit test further supported the model’s adequacy. The lack-of-fit F-value was 1.63 with a *p*-value of 0.3070, indicating that the lack of fit was not significant compared to the pure error. This non-significant result was desirable, implying that the model fitted the data well and that the unexplained variance was likely due to random error rather than model deficiencies.

As for the regression coefficients, the intercept was estimated at 5.95 with a 95% confidence interval ranging from 5.19 to 6.72. The main effect of power had a negative coefficient of −1.51 (CI: −2.56 to −0.47), confirming its strong influence in reducing the response variable. Distance had a positive but non-significant estimate of 0.81 (CI: −0.21 to 1.82), and speed flow had a minor, negative, and non-significant effect at −0.31 (CI: −1.38 to 0.75). The interaction between power and distance (AB) was also negative and significant, with a coefficient of −1.87 and a confidence interval ranging from −3.19 to −0.54. The interactions between AC and BC were not significant, with wide confidence intervals that included zero. All variance inflation factors (VIFs) were close to 1, indicating minimal multicollinearity between predictors.

In summary, the 2FI model was statistically significant overall, primarily due to the effects of power and its interaction with distance. While its ability to predict new data was limited, the model fitted the existing experimental data reasonably well and maintained an adequate signal strength for exploration within the design space.

The results are shown in Figure 11, Figure 12, Figure 13, Figure 14, Figure 15, Figure 16, Figure 17, Figure 18, Figure 19, Figure 20, Figure 21, Figure 22, Figure 23 and Figure 24.

A Box–Cox plot in the context of the Design of Experiments (DoEs) is a visual tool used to identify the most appropriate transformation for the response variable in order to meet the assumptions of statistical modeling, such as normality or constant variance. The plot helps determine if a transformation of the response variable is necessary to improve the model fit and ensure that the results of the experiment are valid.

In a Box–Cox plot, the x-axis represents different values of the transformation parameter, while the y-axis displays the log-likelihood of the response data for each transformation. The plot essentially shows how the likelihood of data changes as the transformation parameter is varied. By observing the plot, the optimal transformation can be identified as the point where the log-likelihood is maximized, indicating the transformation that best normalizes the data.

The plot helps to determine whether a transformation, such as a log transformation or a power transformation, is needed to stabilize variance, make the data more normally distributed, or linearize relationships between variables. If the plot shows that the best transformation occurs at or near a value of zero, this often suggests that a log transformation is most suitable. Conversely, if the optimal transformation occurs at other values of the parameter, it indicates that a different type of transformation may be beneficial.

In the context of DoEs, a Box–Cox plot is particularly useful because it aids in determining the best transformation that ensures valid statistical analysis and improves the accuracy and interpretability of experimental results. It is especially helpful when the response variable exhibits skewness, non-linearity, or unequal variability across levels, which could otherwise lead to unreliable conclusions.

A cube plot is a graphical representation used in the Design of Experiments (DoEs) to visualize the outcomes of an experimental design involving multiple factors. It displays experimental points at the corners of a cube, where each axis of the cube corresponds to a factor being studied, and the points represent different combinations of the factor levels (typically low and high). Each corner of the cube corresponds to a unique combination of the factors at their extreme levels, and the plot helps to visualize how the response variable changes across these combinations. By analyzing the distribution of these experimental points within the cube, one can gain insights into the relationships between factors and their influence on the response (Figure 19).

A cube plot in the context of the Design of Experiments (DoEs) is a powerful graphical tool that helps to visualize the effects of multiple factors on a response variable within a 3D space. It is particularly beneficial for experiments involving three or more factors. The cube plot represents a factorial design, with each factor varied between its low and high levels (often denoted as “−1” and “+1” levels in a two-level design). Each axis of the plot corresponds to one factor, and the corners of the cube represent all combinations of these factor levels. For example, in a three-factor design, there are eight corners, each representing a unique combination of low and high levels for all three factors. The response variable, which represents the measured outcome (such as product yield or mechanical properties), is typically depicted using color or surface contours on the cube. Each experimental point, located at the corners, corresponds to a specific factor combination and the resulting response. The response can be represented by color-coded markers, surface plots, or mesh grids connecting the data points. Cube plots are commonly used in factorial experiments where all combinations of factor levels are tested, and for a two-level factorial design, this means testing all low and high levels for each factor. In a three-factor design, eight combinations (2^3^ = 8) are tested and plotted at the cube’s corners. While higher-order designs with more factors can extend the cube into higher-dimensional spaces, these are typically visualized using projections or slices in two or three dimensions. Cube plots are particularly useful for identifying interactions between factors, which occur when the effect of one factor depends on the level of another factor. Such interactions can be recognized in the plot through non-linear trends or when the response changes unevenly across different combinations of factors. The visual nature of a cube plot allows for the quick identification of interactions, as the response’s behavior is easily observed across factor combinations. By examining how the response varies along each axis, the plot can reveal the main effects of individual factors and the potential presence of interactions. It can also highlight the optimal region in the factor space where the desired outcome occurs, often indicated by specific colors or patterns on the plot. Cube plots are essential for process optimization, as they help identify the factor levels that lead to the best response. They also provide valuable insight into multivariate interactions that may not be evident when examining factors independently.

### 3.3. Optimization of the Electrospinning Process

After a successful model has been verified in the Design of Experiments (DoEs), the next critical step is optimization using response surface methodology (RSM). RSM is employed to identify the optimal settings of the input variables that result in the most desirable outcome for the response variable(s). This involves analyzing the fitted model to explore the relationships between factors and responses, typically using contour plots, 3D surface plots, and numerical optimization tools provided within the DoE software. The goal is to locate the precise combination of factor levels that either maximizes, minimizes, or achieves a target value for the response, depending on the experimental objectives. During this phase, desirability functions may be used when optimizing multiple responses simultaneously, allowing researchers to balance competing objectives. Once the optimal region is identified, validation experiments are usually conducted to confirm that the predicted responses are accurate and reproducible under the recommended conditions. This step not only fine-tunes the process but also provides valuable insights into the robustness and reliability of the optimized system.

**Figure 21:**
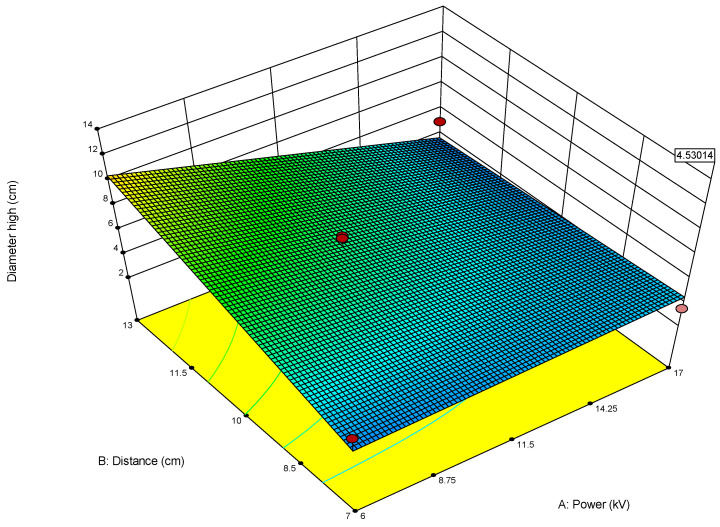
Three-dimensional plot of modeled diameter height in relation to parameters A (power) and B (target distance).

**Figure 22:**
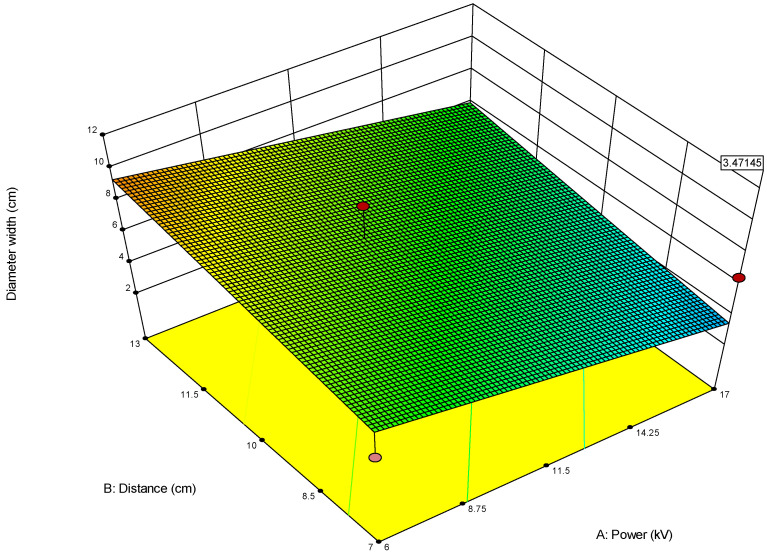
3D plot of modeled diameter width in relation to parameters A (power) and B (target distance).

**Figure 23:**
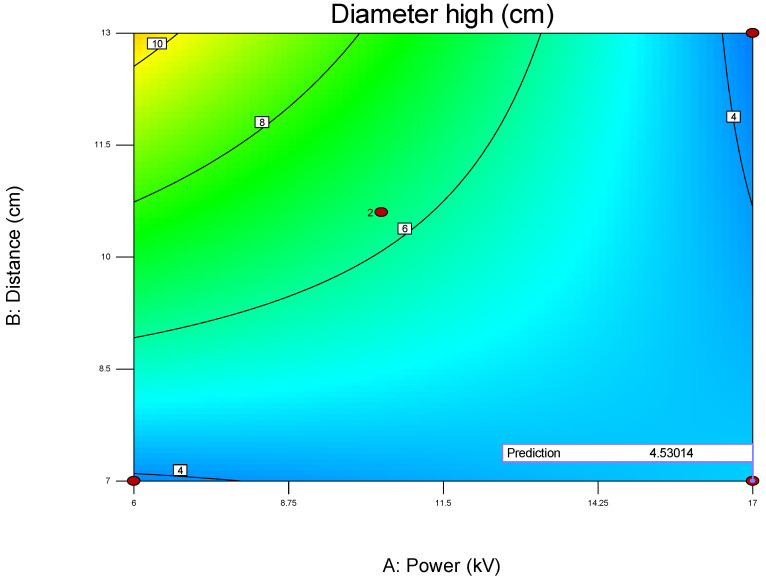
Two-dimensional plot of modeled diameter height in relation to parameters A (power) and B (target distance).

**Figure 24:**
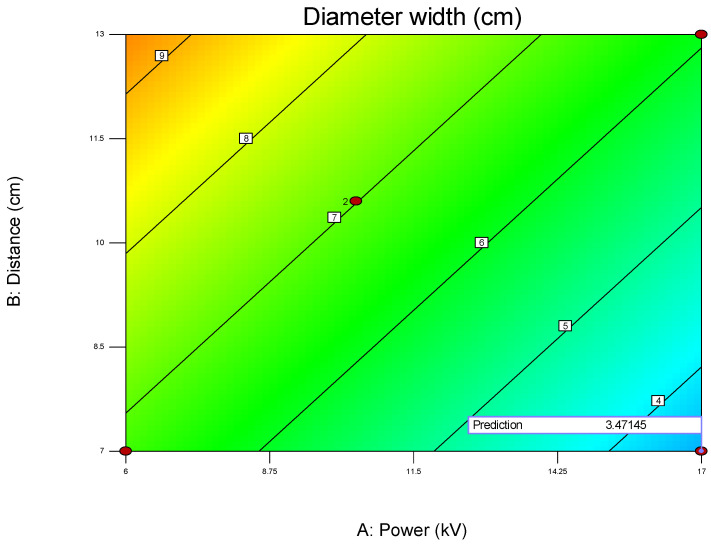
Two-dimensional plot of modeled diameter width in relation to parameters A (power) and B (target distance).

**Figure 25:**
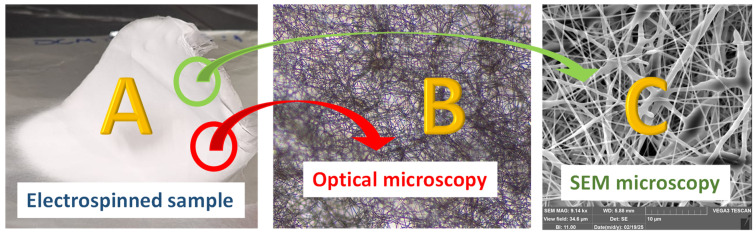
Obtained membrane sample (**A**) polymer material made by electrospinning and (**B**,**C**) microscopic pictures of the nanofibers in the net.

Before optimization, several goals were established: all input parameters were in range (namely, power from 6 to 17 kV, target distance from 7 to 13 cm, and pump speed flow ranging from 10 to 17 mL/min), and the membrane height, as well as the membrane width, were minimal so that the density of the material was maximal. After optimization, several solutions were offered by the response surface methodology, as shown in Table 5 and Figure 21, Figure 22, Figure 23, Figure 24 and Figure 25. Figure 21 and Figure 22 are showing three-dimensional plots of modeled parameters (diameter height and width) in relation to parameters A (power) and B (target distance), and Figure 23 and Figure 24 two-dimensional plots of modeled diameter parameters (height and width) in relation to parameters A (power) and B (target distance).

**Table 5:** Optimal solutions of input parameters in five combinations, with estimated output data and their desirability.

	Input Parameters			Estimated Output Parameters		
Number	Power	Distance	Speed Flow	Diameter Width	Diameter Height	Desirability
1	17.000	7.000	17.000	3.471	4.530	0.812
2	17.000	7.022	17.000	3.481	4.527	0.811
3	17.000	7.001	16.973	3.473	4.537	0.811
4	16.954	7.000	17.000	3.485	4.528	0.811
5	17.000	7.046	17.000	3.491	4.524	0.811

### 3.4. Results of Chemical Characterization of Polymer Membrane

**Figure 26:**
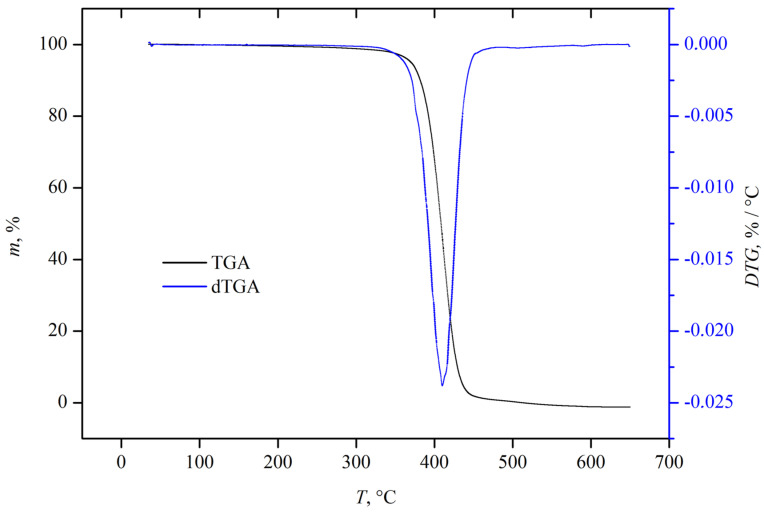
TG and DTG curves of polymer PCL.

The results of the chemical characterization of the obtained membrane are shown in Figure 25 and Figure 26. The sample obtained after electrospinning was recorded under optical and SEM microscopy and afterward investigated using TGA and DSC techniques.

Scanning electron microscopy (SEM) was used to confirm that the fibers obtained by the electrospinning of polycaprolactone (PCL) were indeed nanofibers. As can be seen from Figure 25C, the SEM image provides detailed visual information about the fiber morphology and surface structure. The micrographs show that the fibers are continuous and uniform, with no visible defects such as beads or fused areas. The measured fiber diameters are consistently below one micron, which places them within the nanofiber range. This observation confirms that the electrospinning process successfully produced fibers on the nanoscale. The structural integrity and smoothness of the fibers further support the quality of the fabrication method. Overall, SEM analysis serves as a crucial validation step to demonstrate that the produced fibers met the expected characteristics of nanofibers.

Thermogravimetric analysis (TGA) of the polymer sample polycaprolactone (PCL) provided key thermal stability indicators, including temperatures corresponding to specific mass loss percentages and the temperature at which the maximum rate of mass loss occurs (Figure 26). The temperature at 5% mass loss (T5%) was measured at 375.3 °C, indicating the onset of significant thermal degradation.

The observed thermal degradation onset of the PCL component around 375 °C, which is notably higher than the commonly reported range of ~300–350 °C, suggests an unusual thermal stability that merits closer examination. One plausible explanation for this increased stability could be the presence of additives or stabilizers within the PCL formulation. Additives such as antioxidants, fillers, or nanomaterials are often introduced to improve mechanical properties, processability, or thermal resistance and may shift the degradation onset to higher temperatures by inhibiting chain scission or oxidative degradation mechanisms.

Another important factor that could contribute to this shift is a modification in the polymer’s crystallinity. PCL with a higher degree of crystallinity typically exhibits enhanced thermal stability, as the more ordered crystalline regions require more energy to disrupt than amorphous regions. Processing conditions during synthesis or electrospinning, such as the cooling rate, solvent choice, and thermal history, can significantly influence the crystallinity of PCL. If the sample underwent slow crystallization or post-processing treatments like annealing, this could have led to the formation of more stable crystalline domains, thereby raising the degradation onset temperature. Observed values of the higher degradation temperature of PCL in this case could be the result of a combination of compositional factors (e.g., additives) as well as a combination of structural characteristics (e.g., increased crystallinity).

The temperature at 50% mass loss (T50%) was 411.1 °C, while the temperature at 90% mass loss (T90%) reached 432.3 °C. The temperature corresponding to the maximum rate of mass loss (Tmax) was recorded at 412.6 °C. These values reflect the high thermal stability of PCL and are important for assessing its suitability in temperature-dependent processing and applications. This proves the required thermal stability of the polymer, which is crucial for the electrospinning process. By determining the decomposition temperature of PCL, TGA helped ensure that the processing conditions, such as temperature during solution preparation and fiber collection, remained well below the degradation threshold. This was important for maintaining the integrity and properties of the polymer throughout fabrication. TGA also allowed for the detection of any residual solvents or impurities, which could affect the quality and reproducibility of electrospun fibers.

From the obtained thermograms, indications of the glass transition temperature (Tg) of PCL can be observed around −60 °C. However, for a more pronounced and clearly visible transition, it would be necessary to adjust the measurement conditions, such as modifying the heating rate, applying isothermal steps, or improving baseline stabilization during the analysis.

The electrospinning of polymeric membranes, such as those based on polycaprolactone (PCL), offers a highly tunable method for producing nanofibrous materials with precise control over morphology, porosity, and surface properties. In this study, membranes were fabricated using a PCL solution prepared in a DCM/DMF solvent system and optimized across 22 electrospinning experiments, varying parameters such as voltage, flow rate, and tip-to-collector distance. The resulting membranes were characterized for physical properties, including layer height and width, and subjected to a droplet test that revealed significant differences in absorption time and surface interaction. For instance, lower membrane density was associated with a more pronounced coffee-ring effect, which could be directly correlated with parameters such as porosity and fiber distribution—key findings that emphasize the influence of process conditions on final membrane performance. Complementing these experimental results, thermal analysis (TGA and DSC) provided insight into the thermal stability and crystallinity of the membranes, with degradation beginning at around 375 °C and melting transitions showing variations between first and second heating cycles, indicating structural changes due to processing or interactions with additives. Integrating these data with a life cycle assessment (LCA) approach highlights the importance of developing membranes within a sustainable framework. Electrospun membranes, while technologically promising, must also align with zero-net emissions strategies, particularly through the reduction of solvent waste, energy-efficient production processes, and the selection of biodegradable or bio-based polymers like PCL. The alignment of process optimization through the Design of Experiments (DoEs), combined with robust characterization and LCA, supports the vision of electrospun membranes as functional, scalable, and environmentally responsible components for biomedical and analytical applications, fitting into the broader concept of zero-net technologies aimed at minimizing ecological impact without compromising performance.

The results demonstrate that changes in process conditions significantly influenced the final membrane architecture, as reflected in the droplet absorption tests and the suppression or presence of the coffee-ring effect, which correlated with membrane density and porosity. Thermal characterization through TGA and DSC further revealed that the membranes exhibited high thermal stability, with initial degradation occurring around 375 °C and melting transitions indicating structural consistency and material integrity. These material and process optimizations were evaluated in the broader context of sustainability through a life cycle assessment (LCA), which serves as a key metric in quantifying environmental impacts. Preliminary LCA data indicate that membranes produced from recycled polymers can reduce CO₂ emissions by up to 60–80% compared to conventional production pathways. Moreover, the integration of renewable energy sources, such as solar or wind power, during critical processing stages—alongside closed-loop solvent recovery systems—substantially reduces emissions and resource use, enabling a cleaner, more efficient production model. This integrated, low-impact strategy exemplifies the core goals of zero-waste and net-zero technologies, driving innovation in membrane fabrication while aligning with global environmental targets.

Crucially, this approach represents a meaningful step toward transitioning from a linear to a circular economy within polymer and materials engineering. By incorporating advanced recycling technologies—such as the chemical recycling of post-consumer textile waste into functional biopolymer membranes—the process closes material loops, significantly reduces reliance on virgin petrochemical inputs, and adds value to what would otherwise be environmental burdens [41,42,43,44]. These membranes, sourced from waste streams, are not only functional and high-performing but are also designed for longevity and recyclability.

Recent findings have revealed that membrane curvature is no longer considered a passive consequence of cellular activity but rather an active mechanism for creating membrane domains and organizing centers for membrane trafficking. Curvature can be dynamically modulated through changes in lipid composition, the oligomerization of curvature scaffolding proteins, and the reversible insertion of protein regions that function as wedges within the membrane. An interplay between curvature-generating and curvature-sensing proteins has been observed during vesicle budding. This interaction is also seen in the formation of microenvironments. On a larger scale, membrane curvature plays a crucial role in processes such as cell growth, division, and movement [45,46].

Membrane production needs to follow circular economy principles, including resource recovery, product remanufacturing, and the revalorization of materials that might otherwise end up in landfills. Key life cycle metrics—including carbon footprint, energy and water usage, and toxicity—highlight the environmental advantages of this strategy, especially when renewable inputs and efficient solvent recovery systems are employed. Closed-loop solvent systems and renewable-powered processing steps help reduce VOC emissions, lower energy demand, and cut operational costs while ensuring the long-term sustainability of the manufacturing process. Altogether, this unified strategy of electrospinning optimization, LCA integration, and circular economy alignment demonstrates how polymer membrane technologies can contribute to the future of sustainable, circular, and climate-conscious industrial practices. Moreover, their combination with active components such as antimicrobial nanoparticles or pharmaceuticals [36,37,38,39] may significantly make an impact on the global market. Therefore, our next investigation will be focused on applying active substrates inside polymers in order to obtain functional advanced materials.

Furthermore, addressing the coffee-ring effect—a key challenge in evaporation-driven fabrication—has emerged as essential for achieving uniform membrane structures. Insights into the mechanisms driving solute deposition, including contact line pinning and capillary flow dynamics, contribute to both mitigating defects and exploiting this phenomenon for advanced material assembly. Theoretical models continue to refine control strategies, offering a deeper understanding of pattern formation and enabling tailored membrane design.

On a broader scale, integrating renewable energy, circular design principles, and life-cycle assessments aligns this approach with global sustainability goals. The chemical recycling of textile waste, when combined with electrospinning and zero-waste technologies, not only diverts post-consumer materials from landfills but also redefines value creation in material production. While challenges such as feedstock variability, process scalability, and chemical safety remain, advances in automation, digital design tools, and hybrid membrane development hold promise for overcoming these barriers.

In conclusion, this research contributes to the evolving field of green materials science by demonstrating that waste-derived biopolymers can be engineered into high-functional membranes. With continued innovation and responsible implementation, this approach paves the way for circular, low-emission solutions that align environmental stewardship with industrial transformation.

## 4. Conclusions

This study demonstrates the potential of transforming polymer (for example, textile) waste into high-performance biopolymer membranes through electrospinning, offering a promising pathway toward sustainable material innovation. A critical analysis of the processing parameters reveals that optimal fiber morphology, porosity, and mechanical strength were achieved under specific conditions—namely, 18 kV voltage and 10 wt% polymer concentration in a chloroform/DMF solvent system—and enhanced by the incorporation of a minor cellulose fraction. These membranes displayed high hydrophilicity and selective permeability, underscoring their suitability for biomedical and environmental applications.

The current work, while providing valuable insights into the electrospinning process of polycaprolactone (PCL), could not address all the questions necessary for a comprehensive optimization of the electrospinning system. This study is considered a preliminary experiment, focusing primarily on setting up the initial parameters and identifying key variables that influence the electrospinning outcome. Several aspects, such as the impact of ambient conditions, exact solution properties, and more complex system interactions, were not fully explored. Furthermore, the optimal processing conditions for achieving the desired fiber morphology and functionality still require further investigation. Future work will need to expand upon these findings, refining the setup and testing a broader range of variables to achieve full system optimization.

## Figures and Tables

**Table 1:** Typical applications of different cellulose membranes and their extensive features.

Polymer	Features	Typical Applications
Cellulose acetate	High flow rates, thermal stability, very low non-specific adsorption	Protein filtration, biological and clinical analysis, sterility tests
Surfactant-free cellulose acetate	Excellent wettability, less extractables, very low non-specific adsorption	Removal of particles and microorganisms from aqueous solutions
Cellulose nitrate	Very high protein and DNA binding	Cell retention, buffer filtration, microbiological testing
Regenerated cellulose	Strong chemical resistance, low protein binding	Particle removal from organic and aqueous media, ultracleaning of solutions for HPLC

**Table 2:** Preliminary experiments for Design of Experiments.

	Input Parameters			Output Parameters		
	A Power, kV	B Distance, cm	C Flow, ×10^−3^ mL/min	Height, cm	Width, cm	Coffee-Ring Effect/Drop Test, s
1	6.000	13.000	10.000	10.0	12.5	15.0
2	10.455	7.000	12.835	8.5	6.5	16.0
3	17.000	10.570	12.835	4.5	4.5	14.8
4	12.380	13.000	14.340	6.0	5.0	15.6
5	10.455	7.000	12.835	4.0	2.5	13.4
6	17.000	7.000	17.000	6.5	3.5	11.0
7	10.400	10.600	17.000	9.0	7.0	13.1
8	10.400	10.600	17.000	7.0	7.2	12.9
9	6.000	13.000	14.480	8.5	8.5	13.5
10	9.135	10.300	13.136	9.5	6.0	10.4
11	15.821	10.000	16.285	4.5	3.6	14.4
12	17.000	7.000	10.000	3.0	7.5	13.9
13	17.000	10.570	12.835	4.5	4.7	17.1
14	9.135	10.300	13.136	7.0	7.0	14.7
15	17.000	13.000	17.000	5.5	5.0	13.9
16	6.000	9.100	10.000	7.5	6.5	13.5
17	13.150	13.000	10.000	10.0	5.0	10.7
18	13.150	13.000	10.000	7.0	4.5	16.5
19	6.000	7.000	17.000	5.0	5.0	14.3
20	16.010	7.510	13.500	2.0	6.0	10.8

**Table 3:** Additional experiments for checking the accuracy of the model.

	A Power, kV	B Distance, cm	C Flow, ×10^−3^ mL/min	Height, cm	Height Experiment, cm	Difference, Error, %
21	12,000	12,000	12,000	7.5	7.4	1.3
22	10	10	10	5.5	6.6	16

**Table 4:** Predicted optimal combinations of instrumental parameters for desired samples.

	A Power, kV	B Distance, cm	C Flow, ×10^−3^ mL/min	Height, cm	Width, cm
1	17,000	7000	17,000	3000	2500
2	6000	13,000	17,000	2000	300

## Data Availability

Data are contained within the article.

## Abbreviations

The following abbreviations are used in this manuscript:

DCM	Dichloromethane
DMF	Dimethylformamide
DSC	Differential scanning calorimetry
NP	Nanoparticle
PCL	Polycaprolactone
SEM	Scanning electron microscopy
TGA	Thermogravimetric analysis
